# Transcription factor-binding *k*-mer analysis clarifies the cell type dependency of binding specificities and cis-regulatory SNPs in humans

**DOI:** 10.1186/s12864-023-09692-9

**Published:** 2023-10-07

**Authors:** Saeko Tahara, Takaho Tsuchiya, Hirotaka Matsumoto, Haruka Ozaki

**Affiliations:** 1 Bioinformatics Laboratory, Institute of Medicine, University of Tsukuba, 1-1-1 Tennodai, Tsukuba, Ibaraki 305-8577 Japan; 2 School of Medicine, University of Tsukuba, 1-1-1 Tennodai, Tsukuba, Ibaraki 305-8577 Japan; 3 Center for Artificial Intelligence Research, University of Tsukuba, 1-1-1 Tennodai, Tsukuba, Ibaraki 305-8577 Japan; 4 School of Information and Data Sciences, Nagasaki University, 1-14, Bunkyo-Machi, Nagasaki City, Nagasaki, 852-8521 Japan; 5 Laboratory for Bioinformatics Research, RIKEN Center for Biosystems Dynamics, Wako, Saitama 351-0198 Japan

**Keywords:** Functional genomics, Transcription factor, ChIP-seq, DNA-binding motif, Cell type dependency, *k*-mer-based analysis, Differential *k*-mer analysis, Regulatory SNP, GWAS-SNP

## Abstract

**Background:**

Transcription factors (TFs) exhibit heterogeneous DNA-binding specificities in individual cells and whole organisms under natural conditions, and de novo motif discovery usually provides multiple motifs, even from a single chromatin immunoprecipitation-sequencing (ChIP-seq) sample. Despite the accumulation of ChIP-seq data and ChIP-seq-derived motifs, the diversity of DNA-binding specificities across different TFs and cell types remains largely unexplored.

**Results:**

Here, we applied MOCCS2, our *k*-mer-based motif discovery method, to a collection of human TF ChIP-seq samples across diverse TFs and cell types, and systematically computed profiles of TF-binding specificity scores for all *k*-mers. After quality control, we compiled a set of TF-binding specificity score profiles for 2,976 high-quality ChIP-seq samples, comprising 473 TFs and 398 cell types. Using these high-quality samples, we confirmed that the *k*-mer-based TF-binding specificity profiles reflected TF- or TF-family dependent DNA-binding specificities. We then compared the binding specificity scores of ChIP-seq samples with the same TFs but with different cell type classes and found that half of the analyzed TFs exhibited differences in DNA-binding specificities across cell type classes. Additionally, we devised a method to detect differentially bound *k*-mers between two ChIP-seq samples and detected *k*-mers exhibiting statistically significant differences in binding specificity scores. Moreover, we demonstrated that differences in the binding specificity scores between *k*-mers on the reference and alternative alleles could be used to predict the effect of variants on TF binding, as validated by in vitro and in vivo assay datasets. Finally, we demonstrated that binding specificity score differences can be used to interpret disease-associated non-coding single-nucleotide polymorphisms (SNPs) as TF-affecting SNPs and provide candidates responsible for TFs and cell types.

**Conclusions:**

Our study provides a basis for investigating the regulation of gene expression in a TF-, TF family-, or cell-type-dependent manner. Furthermore, our differential analysis of binding-specificity scores highlights noncoding disease-associated variants in humans.

**Supplementary Information:**

The online version contains supplementary material available at 10.1186/s12864-023-09692-9.

## Background

The regulation of gene expression is one of the most important mechanisms underlying proper cell function. Dysregulation of gene expression results in diseases such as developmental disorders and cancer. Gene expression is regulated by transcription factors (TFs) that bind to DNA by recognizing specific sequences. The human genome is estimated to contain more than 1,600 human TFs, comprising more than 70 DNA-binding domain types [1]. Studies on TF-binding sites have revealed that TFs do not bind to a single sequence, but rather to a distinct set of similar DNA sequences. Such specific sequence patterns are known as DNA-binding motifs. These motifs can be elucidated using several representations, including consensus sequences, position weight matrices (PWMs), *k*-mers, and hidden Markov models [2–4].

Chromatin immunoprecipitation-sequencing (ChIP-seq) is used to detect in vivo TF-binding sites (TFBSs) in a genome-wide manner and generates thousands of DNA sequences with lengths of several hundred base pairs around TFBSs. Several specialized tools have been developed for de novo motif discovery using ChIP-seq data [5]. Usually, more than one motif is found in a single ChIP-seq sample [6]. This reflects the heterogeneous sequence context surrounding TFBSs due to ambiguity in DNA recognition, different binding modes (e.g., heterodimerization, cooperative binding, and tethering), and the existence of transcriptional cofactor motifs. Motifs derived from ChIP-seq and other assays (e.g., protein-binding microarrays and SELEX) have been summarized in several TF motif databases as PWMs or position frequency matrices [7–10].

Despite the accumulation of ChIP-seq data and ChIP-seq-derived motifs, the diversity of TF-binding DNA sequences remains largely unknown. In particular, the differences in TF-binding sequences among different cell types or TFs have not been systematically explored. Many systematic studies using large ChIP-seq datasets have compared the localization and colocalization of TFBSs among cell types and TFs. They have revealed cell type specificities in TFBSs (e.g., [11]) and TF regulatory relationships [12]. Differences in TFBSs for the same TFs in different cell types have been attributed to changes in the TF partner [13, 14] or epigenome [15]. In contrast, several studies have attempted to identify discriminative motifs in a small number of ChIP-seq samples. These studies revealed distinct motifs among homologous TFs [16, 17], cooperative partner TFs [18], or different cell types [19]. However, the extent of diversity in TF-binding sequences across different TFs and cell types remains to be explored.

Recently, ChIP-seq data have been collected in secondary databases [20–23]. These compendiums of ChIP-seq data provide opportunities to analyze the diversity of TF-binding sequences. For this purpose, *k*-mer representation is helpful, because *k*-mer representation can capture low-frequency sequences [24, 25] and has high interpretability [25, 26]. Several methods have been proposed to discover *k*-mer motifs in ChIP-seq data [4, 24, 25, 27–31] and to predict the effect of nucleotide substitutions on TF binding [32]. Thus, a comprehensive analysis and comparison of *k*-mer representations of the TF-binding sequences in each ChIP-seq sample would reveal the diversity of TF-binding sequences among different cell types and TFs within the entire available human TF ChIP-seq dataset. Such approach is exemplified in studies comparing *k*-mer motifs among different TFBSs of the same TF [30] or homologous TFs [17].

**Fig. 1:**
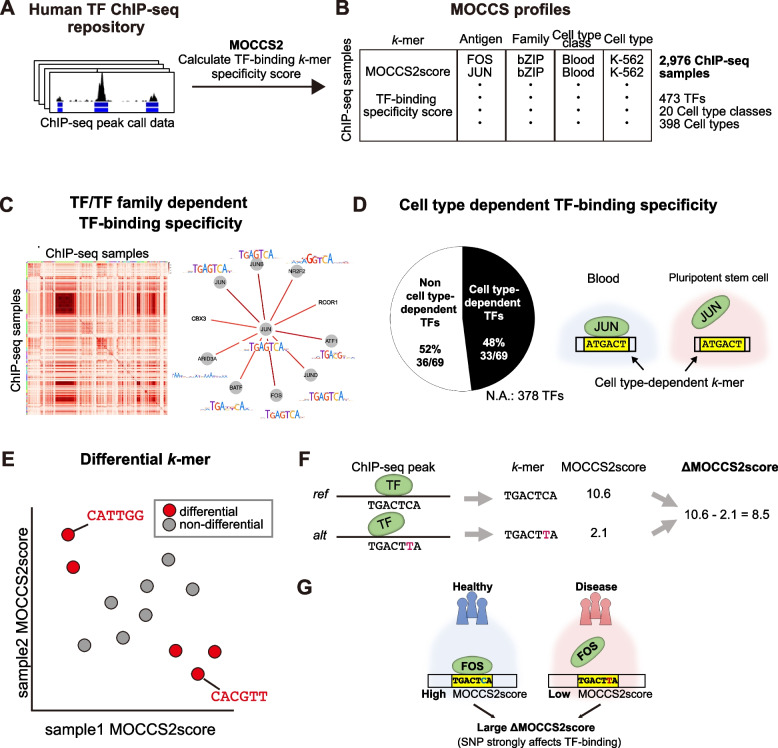
Overview of MOCCS profiles for human TF ChIP-seq samples across TFs and cell types. **A** and** B** Procedure for obtaining MOCCS profiles. Human TF ChIP-seq samples across diverse TFs and cell types were obtained from ChIP-Atlas. Subsequently, MOCCS2, a previously developed *k*-mer-based motif discovery method, was applied to the ChIP-seq dataset. Each ChIP-seq sample was represented as a profile of TF-binding specificity scores (MOCCS2scores) for each *k*-mer sequence, designated as a MOCCS profile. **C** Similarities in MOCCS profiles between ChIP-seq samples were marked by similarities in TFs (TF families), and interactions with other TFs. **D** Comparing the MOCCS profiles for the same TF in different cell type classes showed cell-type-dependent TF-binding specificities. Half of the analyzed TFs exhibited differences in DNA-binding specificity across cell types. For the TFs that we could not perform statistical tests on due to a lack of data, etc., they are marked as Not Applicable (N.A.). **E** Differential *k*-mer detection. Differential analysis of the MOCCS profiles revealed differentially bound *k*-mers between ChIP-seq samples of different cell types or TFs. **F** The ΔMOCCS2score for a single-nucleotide polymorphism (SNP) was defined as the difference in the MOCCS2score between *k*-mers on reference and alternative alleles (ref-*k*-mers and alt-*k*-mers) in a single ChIP-seq. The ΔMOCCS2score was used to predict the effects of the SNP on TF binding, which were validated with the results of in vitro and in vivo assay data. **G**: ΔMOCCS2score can be used to interpret how significant non-coding SNPs from GWAS studies affect the binding of TFs in specific cell types

To investigate the diversity of TF-binding sequences, we applied MOCCS2 [24, 30], a previously developed *k*-mer-based motif discovery method, to ~ 3,000 human TF ChIP-seq samples across diverse TFs and cell types (Fig. 1A). Each ChIP-seq sample was represented as a profile of TF-binding specificity scores (MOCCS2scores) for each *k*-mer sequence, designated as a MOCCS profile (Fig. 1B). We demonstrated that similarities in MOCCS profiles between ChIP-seq samples were marked by similarities in TFs (TF families) and interactions with other TFs (Fig. 1C). By comparing the MOCCS profiles for the same TF in different cell type classes, we found that half of the analyzed TFs exhibited differences in DNA-binding specificity across cell types (Fig. 1D). Moreover, differential analysis of the MOCCS profiles revealed differentially bound *k*-mers between ChIP-seq samples of different cell types or TFs (Fig. 1E). Furthermore, we showed that differences in the MOCCS2scores (ΔMOCCS2scores) of each *k*-mer could be used to predict the effects of variants on TF binding, which were validated with the results of in vitro and in vivo assays (Fig. 1F). Finally, we demonstrated that the ΔMOCCS2score can be used to interpret significant non-coding single-nucleotide polymorphisms (SNPs) as TF-affecting single-nucleotide variants and associate them with candidate TFs and cell types (Fig. 1G). Our study demonstrated that MOCCS profile analysis provides a basis for investigating gene expression regulation and non-coding disease-associated variants in humans.

## Results

### Overview of the MOCCS profile dataset across human TF ChIP-seq samples

**Fig. 2:**
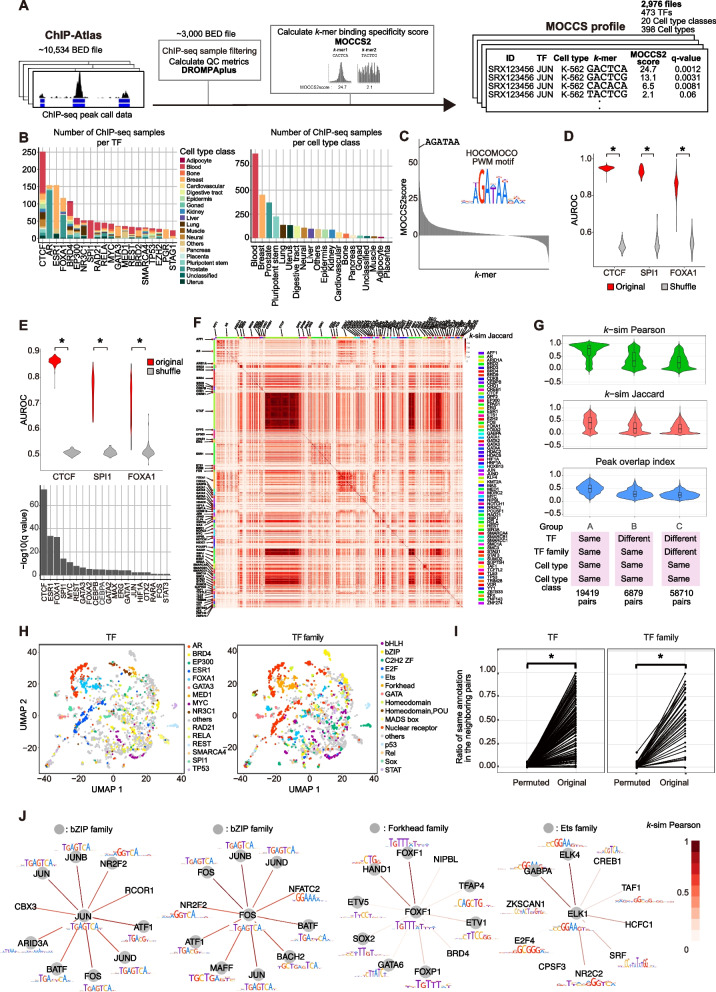
MOCCS profile reflected TF- or TF-family dependent DNA-binding specificities. **A** Overview of the ChIP-seq data processing. MOCCS2 was applied to human ChIP-seq samples from ChIP-Atlas, resulting in MOCCS profiles, *k*-mer-based TF-binding specificity profiles. Quality control metrics for ChIP-seq samples were calculated to filter samples (hard filter). **B** Number of ChIP-seq samples that passed through the hard filter. The colors indicate the cell type class (left) or TF (right). **C** Example of a MOCCS profile (GATA3, MDA-MB231). The highest MOCCS2score *k*-mer (AGATAA) was similar to that of the GATA3 PWM (HOCOMOCO database). **D** Detection performance (AUROC) of canonical motifs (top 10% PWM-supported *k*-mers) using the MOCCS2score for the original (red) and shuffled (gray) data of CTCF, SPI1, and FOXA1. *q < 0.05 (Wilcoxon signed-rank test). **E** Top: Detection performance (AUROC) of significant *k*-mers of MOCCS2 using the top 10% PWM-supported *k*-mers: original (red) and shuffled (gray) data from CTCF, SPI1, and FOXA1. *q < 0.05 (Wilcoxon signed-rank test). Bottom: Bar plot displaying -log10(q-value) from Wilcoxon signed-rank test for 20 TFs. **F** Heatmap of TF-dependent binding *k*-mer similarity (*k*-sim Jaccard) between the ChIP-seq samples. The color labels of rows and columns represent the TFs. **G** Violin plots of *k*-mer similarity indices, *k*-sim Pearson (green) and Jaccard (red), and the peak overlap index (blue) for different groups of ChIP-seq pairs. **H** UMAP visualization of MOCCS profiles. Point colors represent the ChIP-seq samples of the top 15 TFs (left) or TF families (right), with the largest sample size, or the rest (gray). **I** Ratios of neighboring pairs of the same TF (left) or TF family (right) for original and permuted data. * *p* < 6.26e-249 (permutation test; see Methods). **J** Star graphs displaying the TF similarity patterns between query TF (center) and the top 10 TFs with the highest *k*-sim Pearson (edge colors). Circles indicate TFs belonging to the same TF family as the query TF. Avairable PWMs (HOCOMOCO database) are shown

To elucidate the diversity of TF-binding sequences, we compiled a list of 2,976 high-quality human TF ChIP-seq samples and obtained their peak calling results from a ChIP-seq data repository, ChIP-Atlas [22] (Methods, Fig. 2A, Fig. S1). We then applied MOCCS2 [24, 30], our previously developed *k*-mer-based motif discovery tool, to each ChIP-seq sample and quantified the TF-binding specificity of each *k*-mer as a MOCCS2score. As a result, we obtained the profiles of MOCCS2score (MOCCS profiles) for 2,976 high-quality samples across 473 TFs and 20 cell type classes (398 cell types) (Fig. 2B and S1D).

### MOCCS profiles can detect TF-specific binding *k*-mers

We verified whether *k*-mers with a high MOCCS2score represented TF-binding sequences. For example, when MOCCS2 was applied to the GATA3 ChIP-seq sample, the *k*-mer AGATAA, which is a known GATA3 PWM motif, possessed the highest MOCCS2score (Fig. 2C). Furthermore, to confirm that a high MOCCS2score is indicative of TF-binding sequences, we defined the *k*-mer with a top 10% PWM likelihood as a PWM-supported *k*-mer and evaluated the ability of the MOCCS2score to discriminate PWM-supported *k*-mers. Most CTCF (100%), SPI1 (100%), and FOXA1 (89%) ChIP-seq samples exhibited an AUROC exceeding 0.8 (Fig. 2D). These AUROC values were significantly higher than those of the permuted samples obtained by shuffling the MOCCS2scores (Wilcoxon signed-rank test, *p* < 1.2e-125), confirming that the MOCCS profiles can detect PWM-supported TF-binding *k*-mers.

Next, to evaluate the statistical significance of the MOCCS2scores, we calculated the *p*-value of the MOCCS2score for each *k*-mer and the q-value for multiple testing corrections (Methods). We defined a *k*-mer satisfying a q-value < 0.05 as a significant *k*-mer. We verified that the q-values of the MOCCS2score exhibited high performance in the detection of TF-binding sequences (sensitivity > 86.6%, specificity > 99.7%) and effectively controlled for false discovery rate (FDR) using simulated datasets (Fig. S2A, B, and C, Methods). We then detected significant *k*-mers in the real ChIP-seq dataset, and the number of significant *k*-mers in each ChIP-seq sample was correlated with the number of peaks (Fig. S3A and B). In addition, to verify whether significant *k*-mers were consistent with sequences supported by PWMs, we evaluated the ability of PWM likelihood to discriminate significant *k*-mers from other *k*-mers. The AUROCs were significantly higher than those obtained when the PWM likelihood was permuted in 18 of the 20 TFs (Wilcoxon signed-rank test, q-value < 0.05) (Fig. 2E), indicating that significant *k*-mers tend to be supported by PWMs. These results confirm that the MOCCS profiles and significant *k*-mers identified in this study can be used to detect TF-binding *k*-mers in ChIP-seq samples.

### MOCCS profile comparison reveals similarity patterns of TF-binding *k*-mers

Given that MOCCS profiles reflect the binding specificities of TFs, we next attempted to compare the binding specifics between ChIP-seq samples using MOCCS profiles. We obtained the pairwise similarity of binding specificity for each pair of ChIP-seq samples by calculating the Jaccard index (*k*-sim Jaccard) of the two MOCCS profiles (Methods). We found that the same TF exhibited high similarity in the MOCCS profiles (Fig. 2F and G, S4A), which was confirmed when using another similarity metric based on Pearson correlation coefficients (*k*-sim Pearson, Methods) (Fig. 2G and S4A). In addition, ChIP-seq pairs of different TFs from the same TF family (group B) exhibited significantly higher similarities than pairs of different TFs and TF families (group C) (Mann–Whitney U test, *p* < 2.2e-16) (Fig. 2G, S4A), which was consistent with the fact that TFs within the same TF families share the same DNA-binding domains. In addition, when we used Uniform Manifold Approximation and Projection (UMAP) to map ChIP-seq samples in a two-dimensional plane based on the Pearson correlation coefficients of MOCCS profiles (Fig. 2H), ChIP-seq samples of the same TF or TF families were located in close proximity on the UMAP plot, whereas this tendency diminished when sample labels were permuted (Fig. 2I, a permutation test, *p* < 6.26e-249). This is partially explained by the degree of overlap of peak regions (peak overlap index) (Methods) because *k*-sim Pearson or Jaccard significantly correlated with the peak overlap index (one-sample *t*-test of the correlation coefficient, *p* < 4.47e-05) (Fig. S4). However, some ChIP-seq sample pairs exhibited high *k*-sim Pearson or Jaccard values, but the peak overlapping index was low (Fig. S4B), suggesting that not only the overlaps of ChIP-seq peaks themselves, but also the derivation from the same TF and TF family, drive the similarity of TF-binding *k*-mers.

Considering these findings, we hypothesized that *k*-sim Pearson could be used to extract similarity patterns among different TFs. From the TF families with the top 10 adjacency values (Fig. 2I), we selected JUN, FOS, FOXF1, and ELK1. Using each of the four TFs as a query, we extracted the top 10 similar TFs based on *k*-sim Pearson (Fig. 2J). For all four query TFs, most of the top 10 similar TFs belonged to the same TF family as that of the query TF. For example, when FOS and JUN were queried, AP-1 proteins [33] (FOS, JUN, and JUNB) were identified as the top three TFs, demonstrating the ability of *k*-sim Pearson to extract TF similarity patterns. The observed similarity and diversity of MOCCS profiles within TF families are consistent with a previous study demonstrating the similarity and diversity of DNA-binding motifs within TF families [1]. Collectively, the MOCCS profile can reflect TF-specific binding sequences, and these comparisons revealed TF similarity patterns among the TFs and TF family-dependent similarities in the binding sequences.

### MOCCS profile comparison reveals cell type-dependent TFs and TF similarity patterns

**Fig. 3:**
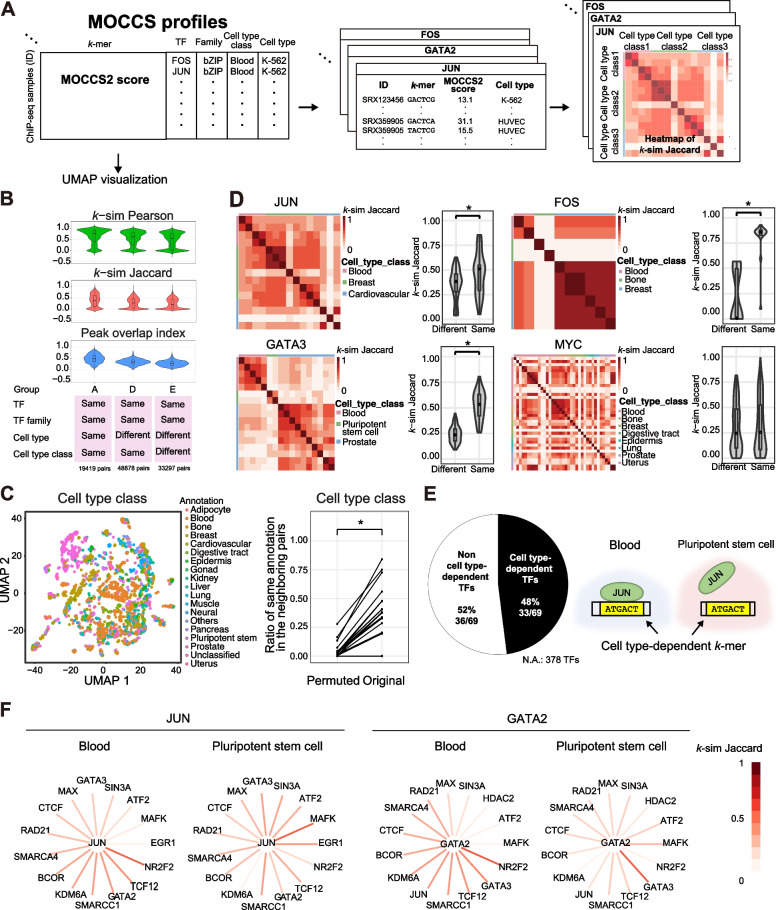
Comparison of MOCCS profiles reveal cell type-dependent TFs and TF similarity patterns. **A** Schematic overview of MOCCS profile comparisons between ChIP-seq samples with the same TF and different cell type classes. **B** Violin plots of *k*-mer similarity indices (*k*-sim), Pearson and Jaccard, and the peak overlap index for different groups of ChIP-seq pairs. **C** Left: UMAP visualization of MOCCS profiles. The point colors represent ChIP-seq samples from different cell type classes. Right: Ratios of neighboring pairs of the same cell type class for the original and permuted data. * *p* < 6.26e-249 (permutation test; see Methods). **D** Heat maps and violin plots of *k*-sim Jaccard values between ChIP-seq samples of the same TFs. The color labels of the heatmaps represent the cell type classes. Cell type classes with only a single ChIP-seq sample were excluded from the visualization. In the violin plots, the x-axis indicates ChIP-seq sample pairs with the same and different cell type classes, and the y-axis indicates *k*-sim Jaccard values. * *p* < 0.05 (Mann–Whitney U test). **E** Left: Pie chart showing the ratio of cell type-dependent to non-cell type-dependent TFs. The null group comprises TFs that could not be tested due to the small sample size. Right: Schematic illustration of the cell type-dependent TFs. For the TFs that we could not perform statistical tests on due to a lack of data, etc., they are marked as Not Applicable (N.A.). **F** Star graphs display cell type-dependent TF similarity patterns for JUN and GATA2. For each query TF (center), the *k*-sim Jaccard value (edge colors) of the query TF and the top 15 TFs with the highest differences in *k*-sim Jaccard values between the two cell type classes (Blood and Pluripotent stem cells) are shown

We further investigated cell type-dependent similarities in MOCCS profiles (Fig. 3A). Based on the annotation matches for TF, TF family, cell type class, or cell type, we divided the MOCCS profiles into three groups with various combinations of annotation matches (Fig. 3B). Compared to group E, groups A and D exhibited a significant increase in the *k*-sim Pearson, *k*-sim Jaccard, and peak overlap indices (Mann–Whitney U test, *p* < 0.001) (Fig. 3B, S5), suggesting cell type-dependent similarities in the MOCCS profiles. We then compared the *k*-sim Pearson and Jaccard with the peak overlap index in each group, revealing significant correlations between *k*-sim Pearson and Jaccard with the peak overlap index (one-sample t-test for the correlation coefficient, *p* < 4.47e-05) (Fig. S5). Accordingly, our *k*-sim Pearson and Jaccard approach quantified the similarities in TF-binding sequences between the two MOCCS profiles, consistent with the peak overlap index. Once again, we performed UMAP visualization and annotated the cell type classes using color, which revealed the adjacency of ChIP-seq samples of the same cell type class (Fig. 3C, left). This tendency diminished when we permuted the cell type class annotations of the ChIP-seq samples, indicating that ChIP-seq samples with the same cell type class had similar MOCCS profiles (Fig. 3C, right). Accordingly, MOCCS profile comparisons revealed cell type-dependent similarities in binding sequences.

Next, we divided the MOCCS profiles for each TF by cell type class and compared the two MOCCS profiles using *k*-sim Jaccard, as it can explicitly quantify the overlaps of significant *k*-mers (Methods) (Fig. 3A, right). For example, JUN exhibited a high *k*-sim Jaccard value in the same cell type class, which was statistically significant compared to the different cell type classes (Fig. 3D). Similarly, we identified cell type-dependent TFs such as FOS and GATA2 and non-cell type-dependent TFs such as MYC (Fig. 3D). Given these examples, we defined cell type-dependent TFs as TFs whose *k*-sim Jaccard exhibited statistical significance between the same and different cell type classes (Mann–Whitney U test, *p* < 0.05). We identified 33 cell type-dependent (48%) and 36 non-cell type-dependent (52%) TFs from the 69 TFs (Fig. 3E, Figs. S6 and 7, Table S1, Table S2). We did not observe any statistically significant preferences on TF families and cell type pairs between cell type-dependent and non-cell type-dependent TFs (FDR > 0.05, the two-sided Chi-squared test for difference of two proportions) (Tables S3 and S4), except for the “Blood-Pluripotency stem cell” pair (FDR = 0.048). Finally, we examined whether cell type-dependent TFs also exhibited cell type-dependent differences in similarity. As a demonstration, we used two cell type-dependent TFs, JUN and GATA2, as queries and extracted the top 15 TFs with large differences in their *k*-sim Jaccard values between the two cell type classes (blood and pluripotent stem cells) (Fig. 3F). We found that cell type-dependent TFs also exhibited differences in similarity to other TFs, despite the availability of ChIP-seq data for the 15 extracted TFs in both cell type classes. For instance, for JUN, the *k*-sim Jaccard with MAFK is higher in Pluripotent stem cells than in Blood. Additionally, for GATA2, the association with NRF2 is greater in Blood than in Pluripotent stem cells. This suggests that cell type dependence in MOCCS profiles may be related to TF cooperation patterns in different cell types. Collectively, these results reveal the cell type dependencies of TF-binding sequences and TF similarity patterns.

### Differentially recognized *k*-mers in two ChIP-seq samples from different cell types or TFs

**Fig. 4:**
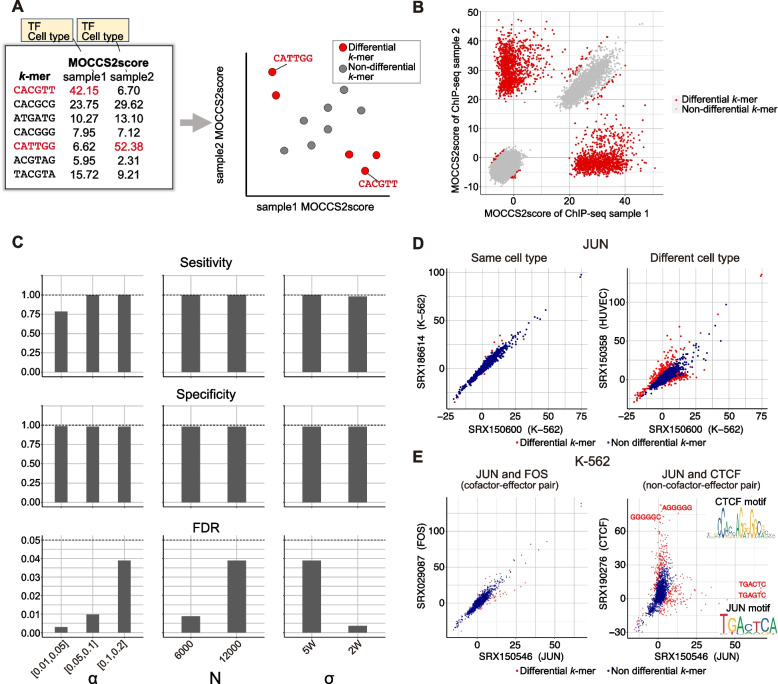
Differential analysis of MOCCS profiles between ChIP-seq sample pairs can detect differentially recognized *k*-mers. **A** Schematic overview of the simulation of differential *k*-mer detection. **B** Simulation results of differential *k*-mer detection. Scatter plot showing MOCCS2scores of all 6-mers in the two simulated ChIP-seq samples. The red and gray points represent the differential *k*-mers (q < 0.05) and other* k*-mers, respectively. **C** Bar plots showing the sensitivity, specificity, and false discovery rate (FDR) of differential *k-*mer detection under different simulation conditions (Fig. S8B). *α* is the percentage of input sequences (ChIP-seq peak regions) containing embedded “true significant *k*-mers,” *N* is the number of peaks in a ChIP-seq sample, and σ is the standard deviation of the embedded “true significant *k*-mers” from the center of the peak. **D** Scatter plots of MOCCS2scores showing differential *k*-mers between two ChIP-seq samples with the same (left) or different (right) cell types for the same TF (JUN). The red and blue points represent the differential *k*-mers (q < 0.05) and other *k*-mers, respectively. **E** Scatter plots of MOCCS2scores showing differential *k*-mers between ChIP-seq sample pairs of different TFs in the same cell types (K-562). The pair JUN and FOS (left) represents cofactor-effector pairs, whereas the pair JUN and CTCF (right) represents non-cofactor-effector pairs. The red and blue points represent differential *k*-mers (q < 0.05) and other *k*-mers, respectively. The PWM-supported differential *k*-mers and known PWM motifs (JASPAR) were compared between JUN and CTCF ChIP-seq samples

Given the sample-level differences in MOCCS profiles among the ChIP-seq samples, we focused on the *k*-mers that exhibited differences in MOCCS2scores between the two ChIP-seq samples. Like differentially expressed genes in RNA-seq analysis, the differential analysis of MOCCS2score would provide “differential *k*-mers,” i.e., *k*-mers that are differentially recognized by TFs between two ChIP-seq samples (Fig. 4A).

To identify differential *k*-mers, we devised a statistical test in which each *k*-mer’s *p*-value was calculated for the difference in the MOCCS2scores between two ChIP-seq samples and the *p*-value was converted to the corresponding q-value for multiple testing correction (Methods). We designated *k*-mers with q < 0.05 as differential *k*-mers. Using the simulated datasets, we verified the q-values of the differential *k*-mers (Fig. S8A and B), resulting in > 75% sensitivity and > 98% specificity and these were controlled for FDR (Fig. 4B and C) under five conditions. This result indicated that our method can reliably detect differentially recognized *k*-mers in two ChIP-seq samples.

When we detected differential *k*-mers between two real ChIP-seq datasets, we found that differences in the biological contexts of the ChIP-seq samples paralleled the number of differential *k*-mers between the two ChIP-seq samples. For example, a comparison of the MOCCS profiles of JUN between the same cell type (K-562) and different cell types (K-562 and HUVEC) identified 10 (0.48%) and 293 (14.0%) differential *k*-mers, respectively (Fig. 4D). This indicated the detection of a higher number of differential *k*-mers in ChIP-seq samples from different cell types than in those from the same cell type. The same tendency applied to the differential *k*-mers between ChIP-seq samples of two different TFs. When we compared the MOCCS profiles of JUN and CTCF in K-562 cells (Fig. 4E), 293 differential *k*-mers (14.0%) were identified, which were greater than the 38 differential *k*-mers (1.82%) identified for the JUN and FOS pairs (Fig. 4E). Because JUN dimerizes with FOS [34], but not with CTCF, the higher number of differential *k*-mers in the comparison of JUN and CTCF is reasonable. For JUN and CTCF, differential *k*-mers with high MOCCS2scores contained PWM-supported *k*-mers (*k*-mers with maximum likelihood from known PWM motifs) (Fig. 4E). These results indicate that differential *k*-mers reflect different TF-binding sequences in different biological contexts such as cell types and TFs.

### ΔMOCCS2score, the difference in the MOCCS2score, illuminates the effects of mutations on TF binding

**Fig. 5:**
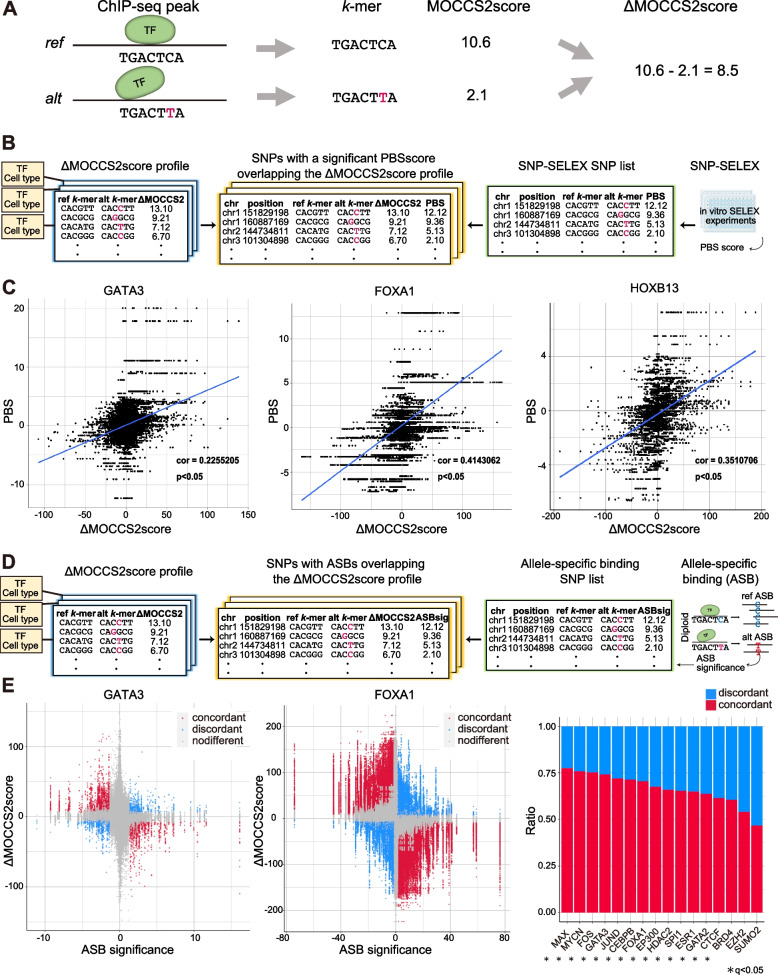
ΔMOCCS2score profiles are consistent with the in vitro SNP-SELEX data and in vivo allele-specific-binding data. **A** Schematic overview of the ΔMOCCS2score calculation for SNP-overlapping TF-binding *k*-mers. **B** Data processing procedures to calculate the ΔMOCCS2score in SNP-overlapping TF-binding *k*-mers for a set of SNPs that exhibited significant differential binding to at least one TF in the SNP-SELEX experiments [35]. **C** Comparison of preferential binding score (PBS) (SNP-SELEX) and ΔMOCCS2score. Each point represents a SNP corresponding to a *k*-mer pair (ref-*k*-mer or alt-*k*-mer). Spearman’s correlation coefficient between the PBS and ΔMOCCS2score and the corresponding *p*-values (one-sample *t*-test) were calculated for each TF. Note that we visualized multiple ΔMOCCS2score values for each SNP in each TF because we calculated ΔMOCCS2scores for multiple ChIP-seq samples of all cell types available for the focal TFs. **D** Data processing procedures to calculate the ΔMOCCS2score for *k*-mers overlapping SNPs with allele-specific-binding (ASB) events [36]. **E** Left and middle: Comparison between ASB significance and ΔMOCCS2score. Each point represents a SNP corresponding to a *k*-mer pair (ref-*k*-mer or alt-*k*-mer). Red points are concordant SNPs and blue points are discordant SNPs. Right: Bar plots displaying the ratios of concordant to discordant SNPs for each TF. Asterisks indicate a significant concordance ratio in the TFs (*p*-values were calculated from the empirical null distribution of the percentage of concordant SNPs and adjusted for multiple testing corrections, q < 0.05)

In our previous studies, we found that differences in MOCCC2scores between *k*-mers associated with known canonical motifs and their variants with 1–2 base substitutions were useful for predicting changes in experimentally measured binding affinities [26, 30]. Based on these observations, we investigated whether the difference in MOCCS2scores between two *k*-mers differing by one nucleotide could indicate single-nucleotide polymorphisms (SNPs) affecting TF binding. Specifically, we defined the ΔMOCCS2score for a SNP as the difference in the MOCCS2score between *k*-mers on reference and alternative alleles (ref-*k*-mers and alt-*k*-mers) in a single ChIP-seq; a positive and large ΔMOCCS2score indicates that a change from the reference to alternative allele potentially attenuates binding of a TF (Methods, Fig. 5A). In this study, we calculated the ΔMOCCS2score for each position within a 6-mer when a SNP was introduced at each respective position.

First, to verify whether the ΔMOCCS2score can be used to evaluate the effect of SNPs overlapping with TF-binding sequences, we compared the ΔMOCCS2score with the results of a high-throughput multiplex protein-DNA binding assay, SNP-SELEX [35] (Fig. 5B). SNP-SELEX uses a preferential binding score (PBS) to assess the influence of SNPs on the in vitro binding specificity of TFs for DNA sequences. A positive and large PBS indicates a change from the reference allele to the alternative allele, strongly attenuating TF binding. We calculated Spearman’s correlation coefficients between the PBS and ΔMOCCS2score of 381–6991 SNPs for 10 TFs. We found that 9 of the 10 TFs exhibited a positive correlation (one-sample *t*-test, *p* < 0.05), whereas a permutation test (100 permutations) by shuffling PBS scores exhibited no correlation (Fig. 5C, Fig. S9A). Furthermore, the SNPs located in the center of the *k*-mers exhibited stronger positive correlations between the PBS and ΔMOCCS2score (Fig. S9B), suggesting that the ΔMOCCS2score correctly detected the effects of the SNPs on TF binding. These results indicate that the ΔMOCCS2score was consistent with the in vitro SNP-SELEX findings.

Next, we compared the ΔMOCCS2score with allele-specific binding (ASB) significance [36], which is a measure of the in vivo ASB of SNPs based on TF ChIP-seq data (Fig. 5D). ASB significance quantifies the influence of SNPs on binding affinity, and a negative and larger ASB significance indicates that a change from the reference allele to the alternative allele potentially attenuates TF binding. We evaluated the fraction of SNPs that were concordant with ΔMOCCS2score and ASB significance (concordant SNPs). For example, 74% of the focal SNPs in the GATA3 ChIP-seq data and 70% in the FOXA1 ChIP-seq data were concordant SNPs (Fig. 5E, left). Among the 16 tested TFs (6864–512,458 SNPs), 14 had significantly higher percentages of concordant SNPs than the permuted negative controls (Fig. 5E, right; Methods), indicating that ASB significance and the ΔMOCCS2score were consistent. Moreover, similar to the consistency between ASB significance and motif fold change from PWM motifs [36], the ΔMOCCS2score was consistent with the motif fold change (note that a negative motif fold change indicates a potential attenuation of TF binding), further supporting the consistency of the ΔMOCCS2score and ASB significance (Fig. S9C). Collectively, these results confirmed that the ΔMOCCS2score of ref-*k*-mers and alt-*k*-mers can infer the effect of SNPs in TF-binding regions on TF-binding.

### Evaluation of GWAS-SNPs in TF-binding regions and prediction of SNP-affected TFs through ΔMOCCS2score profiles

**Fig. 6:**
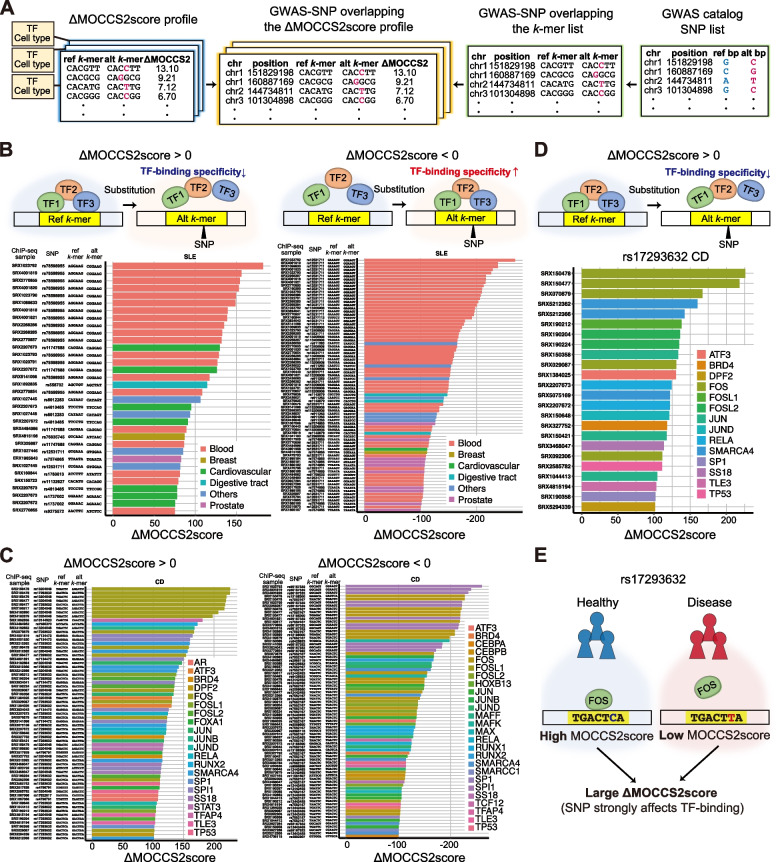
Prediction of effects of GWAS-SNPs on TF binding using ΔMOCCS2score profiles. **A** Schematic overview of the data processing procedures used to calculate the ΔMOCCS2score for *k*-mers overlapping GWAS-SNPs. **B** Combinations of SLE GWAS-SNPs and ChIP-seq samples with high ΔMOCCS2scores (ΔMOCCS2score > 75 (left) or ΔMOCCS2score < –100 (right), q < 0.05). Bar colors represent the cell type classes of the ChIP-seq samples. **C **Combinations of CD GWAS-SNPs and ChIP-seq samples with high ΔMOCCS2scores (ΔMOCCS2score > 100 (left) or ΔMOCCS2score < –100 (right), q < 0.05). Bar colors represent the TFs of the ChIP-seq samples. **D** Prediction of the effect of a CD GWAS-SNP, rs17293632 (C > T), on TF binding using the ΔMOCCS2score profile. The ChIP-seq samples with large positive ΔMOCCS2scores are shown (ΔMOCCS2score > 100, q < 0.05). Bar colors represent TFs. The top three ChIP-seq samples with high absolute values of the ΔMOCCS2score were FOS. **E** GWAS-SNPs predicted to affect FOS binding using ΔMOCCS2score profiles in Crohn’s disease. The CD risk variant, rs17293632 (C > T), may strongly affect the binding of FOS, as shown in **D**

More than 90% of the SNPs reported in genome-wide association studies (GWASs) are located in non-coding regions [37] and are enriched in predicted transcriptional regulatory regions [38]. However, predicting the effect of these SNPs on TF binding remains challenging [39]. To address this issue, we employed the ΔMOCCS2score as a means to infer the effect of each GWAS-SNP on TF binding (Fig. 6A). Four human disease phenotypes were selected: systemic lupus erythematosus (SLE), multiple sclerosis (MS), Crohn’s disease (CD), and inflammatory bowel disease (IBD). Of the 626–971 GWAS-SNPs for each phenotype, 0–24 SNPs overlapped peaks of each ChIP-seq sample (Note that we removed repeat and low-confidence regions in the reference genome), and the GWAS-SNPs with significant ΔMOCCS2scores (q < 0.05, Methods) were distributed in each ChIP-seq sample (Fig. S10).

SLE is an autoimmune disease that affects multiple organs, including the skin, joints, central nervous system, and kidneys [40]. We first focused on the SLE GWAS-SNPs with positive ΔMOCCS2scores (the SNPs that potentially attenuated TF binding) (Fig. 6B, left). SLE GWAS-SNPs exhibited a high ΔMOCCS2score (ΔMOCCS2score > 75) when they overlapped with the peaks in ChIP-seq samples of the blood cell type class, accounting for 63% of the top 30 SNP-ChIP-seq pairs (Fig. 6B, left). In the case of the SNPs with negative ΔMOCCS2scores (the SNPs that potentially enhanced TF binding specificities), SLE GWAS-SNPs exhibited low ΔMOCCS2scores (ΔMOCCS2score < –100) when they overlapped with the peaks in ChIP-seq samples of the blood cell type class, accounting for 84% of the 50 SNP-ChIP-seq pairs (Fig. 6B, right). These results suggest that SLE GWAS-SNPs include SNPs that potentially attenuate and intensify TF binding and are enriched in blood cells, which is consistent with previous reports demonstrating the enrichment of SLE SNPs in chromatin-marked regions specific to hematopoietic cells [41, 42]. Moreover, the SLE GWAS SNPs were enriched in SPI1 ChIP-seq (Fig. S11) and the top GWAS SNPs for SLE corresponded to a known SPI1 motif. These results suggest that the ΔMOCCS2score can be used to predict the cell types in which GWAS-SNPs potentially influence TF-binding specificity.

For CD GWAS-SNPs, ChIP-seq samples of FOS exhibited the highest positive ΔMOCCS2score, which accounted for 43% of the ΔMOCCS2scores of the top 30 SNP-ChIP-seq pairs (Fig. 6C, left). In the case of negative ΔMOCCS2scores, ChIP-seq samples of SPI1 exhibited the lowest ΔMOCCS2score, which accounted for 37% of the ΔMOCCS2scores of the 30 SNP-ChIP-seq pairs (Fig. 6C, right). ChIP-seq samples of FOS also exhibited low ΔMOCCS2scores, which accounted for 40% of the ΔMOCCS2scores of the 30 SNP-ChIP-seq pairs (Fig. 6C, right). While SPI1 and FOS appeared in both positive and negative ΔMOCCS2scores, different SNPs exhibited positive and negative ΔMOCCS2scores, suggesting that CD GWAS-SNPs include SNPs that both potentially attenuate and intensify the binding of FOS and SPI1. Consistently, we found that the alt-*k*-mer of rs56167332 (GGAAGT, ΔMOCCS2score < 0) corresponded to the motif of SPI1, and the ref-*k*-mer of rs13204048 (TGACTC, ΔMOCCS2score > 0) corresponded to the motif of FOS. The enrichment of SPI1 and FOS was also evident in the IBD GWAS SNPs (Fig. S11), which is in line with CD being a type of IBD [43] and a previous report demonstrating fold enrichment of GWAS loci within regions marked for SPI1 binding [44].

Furthermore, to interpret the effects of single CD SNP in detail, we focused on rs17293632 (ref-*k*-mer: GACTCA; alt-*k*-mer: GACTTA), which has been reported as a CD-associated variant and an ASB SNP in JUN and FOS [36, 45]. Among the ChIP-seq samples whose peaks overlapped with rs17293632 with a high ΔMOCCS2score (ΔMOCCS2score > 100), FOS had the top ΔMOCCS2score (ΔMOCCS2score = 226.1) and accounted for 25% of the ΔMOCCS2scores of the top 20 SNP-ChIP-seq pairs (Fig. 6D). In the case of negative ΔMOCCS2scores, there were no SNP-ChIP-seq pairs with a MOCCS2score < –30. These results suggest that rs17293632 can potentially decrease TF-binding (Fig. 6E) and exemplifies how the ΔMOCCS2score can predict which TF binding is altered using a given GWAS-SNP.

Finally, we confirmed that, as the allele frequency of GWAS-SNPs increased, the absolute values of the ΔMOCCS2score and the ratio of SNPs with a significant ΔMOCCS2score tended to decrease (*p* < 0.001 using F-test; Fig. S12). This is consistent with the fact that deleterious alleles tend to have lower allele frequencies in the human population [46]. In summary, the ΔMOCCS2score obtained from the MOCCS profile can be applied to predict combinations of TFs and cell types whose binding specificity is influenced by SNPs associated with human diseases.

## Discussion

In this study, we investigated the diversity of TF-binding sequences by profiling each *k*-mer’s binding specificity across > 10,000 human TF ChIP-seq samples derived from various TFs and cell types using the MOCCS2score. By comparing the MOCCS profiles with conventional PWMs, we confirmed that these profiles could capture *k*-mers recognized by TFs (Fig. 2). We also confirmed that MOCCS profiles capture TF-binding sequence similarities between (1) TFs of the same TF families and (2) cell types of the same cell type classes (Figs. 2 and 3). Moreover, by comparing the MOCCS profiles among the ChIP-seq data of the same TFs and different cell type classes, we found that approximately half of the TFs exhibited cell type dependency in TF-binding sequences (Fig. 3). Cell type-dependent TFs may pose challenges in the use of machine learning to predict TFBSs [47] and require more sophisticated methods such as multi-task learning [48]. In addition, differential *k*-mer analysis revealed that *k*-mers changed the TF-binding specificities between different TFs and cell types (Fig. 4).

Considering that MOCCS profiles represent the DNA-binding specificities of ChIPed TFs in ChIP-seq samples, we used these profiles to predict the impact of SNPs on TF binding. To this end, we calculated ΔMOCCS2scores to quantify the differences in TF-binding specificity between two *k*-mers from the MOCCS profiles. Using in vitro SNP-SELEX and in vivo ASB datasets, we confirmed that the ΔMOCCS2score analysis accurately predicted the SNPs affecting TF binding (Fig. 5). Furthermore, we examined the ΔMOCCS2scores for GWAS-SNPs associated with several diseases across the entire high-quality human ChIP-seq dataset and identified candidate TFs and cell types associated with each disease (Fig. 6). Collectively, these results demonstrate how the MOCCS profiles and ΔMOCCS2scores contribute to our understanding of TF-binding sequences.

In this study, we did not investigate the molecular basis of the cell type dependency of binding sequences. One possibility is that different TF partners alter binding specificity, as systematically investigated using in vitro assays [49] and systematic data analyses [1]. We addressed this possibility by comparing MOCCS profiles with TFBS colocalization patterns. Another possible mechanism is a change in chromatin accessibility and 3D chromatin structure, which are associated with cell type-specific gene expression [50]. This mechanism can be examined by comparing MOCCS profiles with chromatin accessibility and structures using DNase I-seq, ATAC-seq, and Hi-C data.

The value of *k* is a hyperparameter in *k*-mer-based methods, including MOCCS. Determining the appropriate *k* value for each TF remains challenging. In this study, we chose a *k* value of 6, based on the initial examination of the accuracy of detecting PWM canonical motifs as follows: Briefly, we ran MOCCS with k = 6, 7, and 8, calculated the AUROC, as displayed in Fig. 2D, and compared the accuracy of the different* k* values. The results demonstrated that the AUROC decreased as *k* increased from 6 (Fig. S13). Accordingly, we set *k* = 6 for all analyses in this study.

There are several possible directions for future studies in this field. The first is to investigate the relationship between *k*-mer usage and other genomic features, including chromatin states, gene density, and gene function. The second is to use the ΔMOCCS2score to interpret various types of mutation information, including mutation signatures [51] and indels [52]. The third is to apply the ΔMOCCS2score analysis of GWAS-SNPs to drug-target discovery by searching for SNPs affecting TF binding and candidate cell types responsible for the phenotypes [53]. The fourth is to investigate the diversity of TF-binding sequences among human TF homologs, particularly in relation to their functional diversification. For example, the functional diversification of TF homologs was parallel to the diversification of MOCCS profiles in zebrafish [17]. The fifth is to systematically investigate the interposition dependencies within TF-binding motifs across cell types and TFs using the ΔMOCCS2score. Interposition dependencies are not limited to directly adjacent nucleotides [54], and *k*-mer-based motif analyses have revealed interposition dependencies in TF-binding motifs in a limited number of cell types or TFs [26, 30]. The sixth is the use of other motif representations. In this study, we proposed a *k*-mer method to comprehensively analyze binding specificities across various TFs and cell types. Clearly, by integrating other representations such as PWMs and hidden Markov models, there is potential to understand binding specificities in greater detail. Further advancements in this area of research are anticipated. Finally, to help researchers investigate gene expression regulations and human genetics, we are now developing a user-friendly database of the pre-computed results of MOCCS analyses, including MOCCS profiles and ΔMOCCS2scores, for quality-filtered human ChIP-seq samples.

## Conclusions

Our study profiled *k*-mer-based TF-binding specificities for a large-scale dataset of human TF ChIP-seq samples and revealed cell-type-dependent DNA-binding specificities for half of the analyzed TFs. We demonstrated that MOCCS profiles and the ΔMOCCS2score could predict the effects of variants on TF binding and interpret non-coding GWAS-SNPs, providing a basis for investigating gene expression regulation and non-coding disease-associated variants in humans.

## Methods

### Sample filtering of ChIP-seq data

To control the quality of the ChIP-seq samples and MOCCS profiles, quality metrics were obtained and two thresholds for the metrics were set: soft and hard filters. To thoroughly evaluate the quality of ChIP-seq samples, both peak calling and read alignment information were necessary. Therefore, the following steps were performed first. FASTQ files were obtained using sra-tools_2.11.0. sif fasterq-dump or downloaded from the DDBJ database, and FASTQ files with the same SRX ID were concatenated into one FASTQ file. Bowtie2 (version 2.2.5) was then used to convert the FASTQ files to SAM files, which were subsequently converted to BAM files using SAMtools (version 1.9).

#### Soft filter

For the soft filter, quality control metrics were obtained from processing logs in ChIP-Atlas (https://github.com/inutano/chip-atlas/wiki#tables-summarizing-metadata-and-files/) and the read alignment rate was obtained from the bowtie2 results. The thresholds were set as follows: the number of mapped reads, 10,000,000; the number of peaks, 100; and the read alignment rate, 54.09364 (determined by mean − 2SD).

#### Hard filter

For the hard filter, quality metrics from DROMPAplus [55], which is a quality control tool for ChIP-seq experiments, were used. DROMPAplus (version ​​1.8.1) was then applied to the BAM files of the ChIP-seq samples. 10,534 DROMPAplus output files containing five parameters for ChIP-seq quality control were obtained: library complexity, number of mapped reads, GC content, normalized strand cross-correlation coefficient (NSC), and background uniformity (Bu). The thresholds for the hard-filtered samples were set as follows: library complexity > 0.8; number of mapped reads > 10,000,000; GC content < 60; NSC > 2.0; Bu > 0.8; and number of peaks > 100. In addition, the ChIP-seq samples of GFP, epitope tags, BrdU, and biotin were removed.

After applying the soft filter to the initial set of 10,534 human TF ChIP-seq samples provided by ChIP-Atlas, we retained a total of 9,283 ChIP-seq samples (88.1%) (Fig. S1A). However, when we evaluated the performance of the MOCCS2score for distinguishing *k*-mers supported by PWM (top 10% likelihood), some soft-filtered samples still exhibited low performance (< 0.85 area under the receiver operating characteristic curve; AUROC) (Fig. S1B), possibly because of the presence of low-quality ChIP-seq samples. To further remove low-quality ChIP-seq samples, we applied the hard filter and retained 2,976 samples (Fig. S1A and C).

### Preprocessing of ChIP-seq data for MOCCS2

The peak calling data of human TF ChIP-seq samples (hg38) were obtained from the ChIP-Atlas database (https://chip-atlas.org/), each peak region in the BED files was trimmed to + / − 350 bp from the TFBSs (the center of each peak region), and the BED files were converted to FASTA files using the BEDTools (version v2.27.1) getfasta tool for application to MOCCS2 with the option "–mask –low-count-threshold -1", as the former option ignores repeat-masked regions in the genome. Annotations of TFs (antigens), cell types, and cell type classes were also obtained from the ChIP-Atlas database.

### Calculation of the MOCCS2score using MOCCS2

MOCCS2 clarifies TF-binding *k*-mers from ChIP-seq peak calling data, as previously described [24, 30]. Specifically, considering a histogram displaying the appearance of a *k*-mer around TFBSs, MOCCS2 quantifies the sharpness of the histogram for each *k*-mer as an area under the curve (AUC) score and then calculates the MOCCS2score for each *k*-mer by normalizing the AUC scores. The AUC score can be described as the area under the cumulative relative frequency curve of the appearance of each *k*-mer sequence against the distance from the TFBS. $W$ is the size of the analyzed window where *k*-mer sequences are sought around the ChIP-peak positions, and $n$ is the number of *k*-mer appearances in the ChIP-seq samples. If $f(x)$ is the appearance count of each *k*-mer sequence at the position $\pm x$ bp $(x\in [1,W])$ away from the TFBS, then the cumulative relative frequency distribution $F(x)$ for the *k*-mer sequence is calculated as follows:

1 $$F(x)=\frac{{\Sigma }_{i=1}^{x}f(i)}{{\Sigma }_{j=1}^{W}f(j)}$$

and its AUC score is calculated as follows:

2 $$AUC\;score=\Sigma_{x=1}^W(F(x)-\frac xW)$$

The AUC increases as the shape of the histogram becomes sharper.

Some irrelevant *k*-mers with low appearance counts demonstrate high AUC scores due to the large standard deviations (SDs) of the AUC scores for low-occurrence *k*-mers [30]. To compensate for falsely high AUC scores, the MOCCS2score for each *k*-mer was defined as the AUC score divided by the SD at its appearance count. The SD of the AUC score for a *k*-mer was calculated as $\frac{W}{\sqrt{12n}}$, where $n$ is the appearance count of the *k*-mer derived in [30]. The MOCCS2score was calculated as follows [30]:

3 $$MOCCS2score=\frac{\sqrt{12n}}W\lbrack AUC\;score\rbrack$$

### P-value of the MOCCS2score by MOCCS2

The *p*-values of the MOCCS2scores were calculated as follows: When a *k*-mer randomly appears within $\pm W$ bp of a TFBS, $f(x)$ (the distribution of the *k*-mer position from the TFBS) follows a uniform distribution $U(0,W)$ [24, 30]. Hence, the AUC score is regarded as the sample mean of the uniform distribution $U(0,W)$ minus $\frac{W}{2}$:

$${x}_{i}\sim U(0,W) (i=1,...,n)$$

$$AU{C\;score}_{random}=\frac1n\Sigma_{i=1}^nx_i-\frac W2$$

According to the central limit theorem, when $n$ is sufficiently large, the sample mean of $U(0,W)$ follows a normal distribution $N(\frac{W}{2},\frac{{W}^{2}}{12n}$) because the mean and variance of $U(0,W)$ are $\frac{W}{2}$ and $\frac{{W}^{2}}{12}$, respectively. Because the AUC score is the sample mean of $U(0,W)$ subtracted by $\frac{W}{2}$, when *n* is sufficiently large, the AUC score approximately follows a nominal distribution $N(0,\frac{{W}^{2}}{12n})$. The *p*-values of the observed AUC score *s* were calculated as follows:

$$p=1-{\int }_{-\infty }^{s}\frac{1}{\sqrt{2\pi \frac{{W}^{2}}{12n}}}exp\left(-\frac{{x}^{2}}{2\frac{{W}^{2}}{12n}}\right)dx$$

In this study, the *p*-value of the MOCCS2score of a *k*-mer was defined as the *p*-value of the corresponding AUC score.

The *p*-value of the AUC score was evaluated by comparing it with empirical *p*-values based on a simulation experiment. In this process, each *k*-mer position relative to the peak center was simulated by sampling from $U(0,W)$. The AUC was calculated as the sampled mean − W/2, and $n=100$ (assumed a *k*-mer with 100 counts) and $W=250$ were set. The simulation was repeated 10,000 times and the empirical distribution of the AUC score was obtained. The ratio of the empirical standard deviation to the theoretical value was 1.0064, indicating that the *p*-values based on the central limit theorem and those calculated from the empirical cumulative distribution based on the simulation results were roughly consistent.

### Detection and evaluation of significant *k*-mers

Significant *k*-mers were defined as follows: The p-values of the MOCCS2scores of *k*-mers were calculated for each sample. Then, the corresponding q-value was calculated for each sample using the “p.adjust” function in the “stats” package in R for multiple testing corrections. *k*-mers with q < 0.05 were considered significant *k*-mers.

To evaluate the effectiveness of the significant *k*-mers, the classification performance of the significant *k*-mers was predicted using the PWM likelihoods of the ChIP-seq samples of the focal TF and the AUROC was calculated. Permutated samples were also generated by shuffling the PWM likelihood for *k*-mers.

### Evaluation of the prediction performance of PWM-supported *k*-mers based on the MOCCS2score

#### Calculation of the likelihoods for each *k*-mer based on PWM motifs

Motif PWMs were downloaded from the HOCOMOCO database (https://hocomoco11.autosome.ru/downloads_v11). The likelihood of PWM was calculated for each *k*-mer by multiplying each base probability across positions in the PWM. For each *k*-mer and PWM, the *k*-mer was shifted to all possible offsets with respect to the PWM, the likelihood of each offset was computed, and the maximum likelihood value was selected as the representative value among all offsets.

#### Evaluation of the performance of the MOCCS2score to detect PWM-supported *k*-mers

*K*-mers with a high likelihood (top 10%) were defined as “positive” *k*-mers (PWM-supported *k*-mers) and the other *k*-mers as “negative” *k*-mers. The classification performance of the “positive” *k*-mers was evaluated using the MOCCS2score of the ChIP-seq samples of the focal TF and the AUROC was calculated. Permutated samples were generated by shuffling the MOCCS2scores for *k*-mers.

### MOCCS profile comparison

#### Calculation of the *k*-sim Pearson and Jaccard, and peak overlap index

Two similarity indices, *k*-sim Pearson and *k*-sim Jaccard, were defined and calculated for each pair of MOCCS profiles that passed through the hard filter (Fig. 3A). The *k*-sim Pearson of a pair of MOCCS profiles was defined as the Pearson correlation coefficient after setting the MOCCS2scores of the non-significant *k*-mers to zero. Note that MOCCS profiles in which all *k*-mers were non-significant were excluded, as shown in Figs. 2 and 3, Fig. S4, S5, S6, and S7. The *k*-sim Jaccard of a pair of MOCCS profiles was defined as the Jaccard index of two sets of significant *k*-mers in the two MOCCS profiles (q < 0.05). Note that *k*-sim Pearson quantifies the similarity and considers the value of the MOCCS2score of each significant *k*-mer, whereas *k*-sim Jaccard quantifies the degree of overlap of the significant *k*-mers.

The peak overlap index was calculated based on the ChIP-seq peak positions in the BED files obtained from ChIP-Atlas [22], which directly reflects the degree of peak overlap regions. First, for a pair of ChIP-seq samples (indexed as 1 and 2), *n1all* and *n2all* were calculated as the total number of peaks for ChIP-seq samples 1 and 2, respectively. Second, *n1* (*n2*) was counted as the number of peaks in ChIP-seq sample 1 (2) that overlapped with peaks in ChIP-seq sample 2 (1) using BEDTools with the intersect option (intersect -u -a -b) [56]. Finally, the peak overlap index was calculated as follows:

$$peak\;overlap\;index=\frac12\left(\frac{n1}{n1all}+\frac{n2}{n2all}\right)$$

To validate the *k*-sim Pearson and Jaccard indices, they were compared with the peak overlap index (Fig. S4). Note that CTCF was excluded from Figs. 2 and 3, S4 and S5 for visualization. In the grouping of the MOCCS profile pairs, the pairs in which either sample annotation included “Unclassified”, “Others,” or “No annotation” were also excluded.

#### UMAP visualization of MOCCS profiles and statistical tests

UMAP was performed on the set of MOCCS profiles using the R package “umap” [57] with the metric set as “pearson” and a spread of 10. The ChIP-seq samples on the UMAP plot were colored according to the TF, TF family, or cell type class. Unknown pairs whose annotations included “Unclassified”, “Others”, or “No annotation” were excluded.

The ratio of the same annotations (TF, TF family, and cell type class) was calculated in the top three neighboring ChIP-seq sample pairs defined by the *k*-sim Pearson method, and the same annotation ratio was subsequently averaged across the ChIP-seq samples. A permutation test was also performed by (1) shuffling the annotation for the ChIP-seq samples, (2) calculating the same annotation ratio for each ChIP-seq sample, (3) calculating the average of the same annotation ratios across the ChIP-seq samples, and (4) repeating steps (1)–(3) 1,000 times to obtain an empirical null distribution of the same annotation ratio (Figs. 2I and 3C). CTCF was excluded from these UMAP procedures (Figs. 2 H, I and 3C).

#### Evaluation of TF similarity patterns using the *k*-sim Pearson

The *k*-sim Pearson was calculated among the different types of TFs in a cell type class. JUN in the blood, FOS in the blood, FOXF1 in the digestive tract, and ELK1 in the uterus were selected as query TFs, and the *k*-sim Pearson was calculated among the TFs whose ChIP-seq samples passed the hard filter. The TFs with *k*-sim Pearson values in the top ten for each query TF were extracted and visualized as star graphs.

#### Evaluation of TF-dependent similarity of MOCCS profiles using the *k*-sim Jaccard

The *k*-sim Jaccard was calculated for all pairs of MOCCS profiles. These *k*-sim Jaccard values were visualized as heat maps (Fig. 2F). In the heat map matrix, rows and columns represent each ChIP-seq sample. The samples were ordered by TFs, and the color labels were separated by TFs. All cell type classes with only a single ChIP-seq sample were excluded from visualization. Subsequently, TFs for which ChIP-seq samples were from a single cell type class were also excluded.

#### Evaluation of cell type-dependent TFs using the *k*-sim Jaccard

*K*-sim Jaccard values were calculated for all pairs of MOCCS profiles for each TF. These *k*-sim Jaccard values were then visualized as heat maps grouped by cell type class. These *k*-sim Jaccard values were also visualized as violin plots by dividing ChIP-seq pairs into the same or different cell type classes. All cell type classes using only a single ChIP-seq sample were excluded (Fig. 3D and E, Fig. S6 and S7). In addition, the Mann–Whitney U test was used to examine the statistical significance of the differences in the *k*-sim Jaccard values between the same and different cell type class groups. When a TF exhibited a significant difference in the Mann–Whitney U test, it was denoted as a cell type-dependent TF. The Mann–Whitney U test was also performed among all TFs to determine the ratio of cell type-dependent to non-cell type-dependent TFs.

Among the cell type-dependent TFs, JUN and GATA2 were selected as query TFs to compare the TF similarity patterns between the two cell type classes. The 15 TFs with the largest difference in the *k*-sim Jaccard value between the two cell type classes were selected, and the two cell type classes with the highest number of available TFs were also selected.

### Differential k-mer detection between ChIP-seq samples

#### Algorithm of differential *k*-mer detection

To detect *k*-mers that are differentially recognized between two samples, differential *k*-mers were defined as follows: $W$ is the size of the search window for *k*-mer occurrences around TFBSs. ${n}_{i}$ and ${n}_{j}$ are the numbers of appearances of *k*-mers $i$ and $j$, respectively. When *k*-mers $i$ and $j$ appear randomly around the TFBS, the AUC scores of $i$ and $j$ follow normal distributions $N(0, {W}^{2}/12{n}_{i})$ and $N(0, {W}^{2}/12{n}_{j})$, respectively. The difference in the AUC scores between the two *k*-mers can be regarded as the difference in the means between two normally distributed populations with unequal variance. In such cases, a two-sample z-test is applied [58], which tests the hypothesis that two normally distributed populations with unequal variances have equal means. If ${{\sigma }^{2}}_{i}$ and ${{\sigma }^{2}}_{j}$ are the variances in each *k*-mer distribution, and we assume that the variance of the AUC score is constant regardless of the value of the AUC score, the test statistics are

$$Z=\frac{AU{C}_{i}-AU{C}_{j}}{\sqrt{{{\sigma }^{2}}_{i}/1+{{\sigma }^{2}}_{j}/1}}=\frac{AU{C}_{i}-AU{C}_{j}}{\sqrt{{{\sigma }^{2}}_{i}+{{\sigma }^{2}}_{j}}}$$

and exhibit a standard normal distribution. The difference in the AUC scores of the two *k*-mers $AU{C}_{i}-AU{C}_{j}$ followed the normal distribution $N(0,\sqrt{{{\sigma }^{2}}_{i}+{{\sigma }^{2}}_{j}})$. This approach was also applied to the statistical testing of the ΔAUC score (difference in AUC scores between two samples), and *p*-values were calculated from the normal distribution $N(0,\sqrt{{{\sigma }^{2}}_{i}+{{\sigma }^{2}}_{j}})$.

#### Simulation of differential *k*-mer detection

To validate the differential *k*-mer detection method, simulated ChIP-seq peak data were generated (Fig. S8A). Two random ChIP-seq samples (S1 and S2) with $N$ peaks were generated, each of which was a random sequence of length $2W+1$. All *k*-mers were then randomly assigned to one of the three categories (A, B, or C) and the *k*-mers of A and B were embedded in random sequences as follows:

- A: *k*-mers that are deemed as significant *k*-mers in S1 and S2, and non-differential *k*-mers.
- B: *k*-mers that are deemed as significant *k*-mers in either S1 or S2, and differential *k*-mers.
  - B1: k-mers that are deemed as significant k-mers in S1, non-significant k-mers in S2, and differential k-mers that are more bound in the S1 condition.
  - B2: *k*-mers that are deemed as non-significant *k*-mers in S1, significant *k*-mers in S2, and differential *k*-mers that are more bound in the S2 condition
- C: *k*-mers not assigned to A or B

When each of the *k*-mers of A, B1, and B2 were embedded in S1 and S2, a peak (sequence) was first randomly selected, and then the position in the sequence was randomly selected following Gaussian distributions for significant *k*-mers or uniform distributions for non-significant *k*-mers (see Fig. S8A). After all of the *k*-mers were embedded, MOCCS2 was applied to each of the ChIP-seq samples (S1 and S2), and the *p*-value of the difference in the MOCCS2score was calculated.

This simulation encompassed several parameters: *α*, *N*, *σ*, *W*, *l*, *m* (see Fig. S8B). Notably, *m* (number of embedded significant *k*-mers) was set to 90 based on the average number of significant *k*-mers in 100 randomly-selected real ChIP-seq samples. In addition, *l* (number of embedded differential *k*-mers) was set to 45 based on the average number of *effective* differential *k*-mers in the following examination. First, for each pair of 100 randomly-selected real ChIP-seq samples, we (1) detected the differential k-mers, (2) searched for 1-bp- or 2-bp-shifted *k*-mers of the *k*-mers with lower FDR, (3) counted the number of differential *k*-mers after excluding the 1-bp- or 2-bp-shifted *k*-mers ( we call the remaining differential *k*-mers as *effective* differential k-mers). Then, we averaged the number of *effective* differential *k*-mers after excluding the 1-bp- or 2-bp-shifted *k*-mers across the ChIP-seq samples pairs, which was around 45.We employed the number of *effective* differential *k*-mers in the real ChIP-seq samples because we focused on the performance of detecting “true differential *k*-mers”, not the 1-bp- or 2-bp-shifted *k*-mers of them in the simulated ChIP-seq samples.

### Calculation and evaluation of ΔMOCCS2scores for SNPs in a single ChIP-seq sample

#### Preparation of SNP-overlapping *k*-mer lists and calculation of ΔMOCCS2scores

The ΔMOCCS2score for a SNP in a ChIP-seq sample was calculated as follows: First, *k*-mers overlapping SNPs were obtained from the reference genome (hg38) (the SNP sets are described below). Then, *k*-mers corresponding to the reference genome sequence were defined as ref*-k-*mers, and *k*-mers that replaced the reference allele with the alternative allele were defined as alt*-k*-mers, generating pairs of ref-*k*-mers and alt-*k*-mers for SNPs. There are *k* possible positions for SNPs in the *k*-mer; therefore, the positions of the SNPs in the* k*-mer were shifted from the 1st to the *k*th position from the left of the *k*-mer, creating *k* different pairs of ref-*k*-mers and alt-*k*-mers for each SNP.

Next, a table of AUC scores, counts, and MOCCS2scores corresponding to both the ref-*k*-mer and alt-*k*-mer were compiled from the *k*-mer list. The ΔMOCCS2scores (differences of MOCCS2score for ref-*k*-mer and alt-*k*-mer) and their *p*-values and q-values were calculated as in the differential *k*-mer detection algorithm and a ΔMOCCS2score profile was generated for each ChIP-seq sample.

#### Comparison of the ΔMOCCS2score with the SNP-SELEX results

SNP-SELEX results were obtained from GSE118725 [35] and the genomic coordinates of the SNPs were converted from hg19 to hg38 using liftover (https://genome.ucsc.edu/cgi-bin/hgLiftOver) [59]. Then, SNPs overlapping the ChIP-seq sample peak region were selected and the *k*-mers overlapping SNPs from the reference genome were obtained (hg38) (ref-*k*-mer). Subsequently, ref-*k*-mer and alt-*k*-mer pairs were created, the AUC score, count, and MOCCS2score for each *k*-mer was obtained from the MOCCS profile, and the ΔMOCCS2scores, *p*-values, and q-values were calculated for each ChIP-seq sample. SNP-SELEX quantifies the difference in TF-binding specificity between reference and alternative alleles for each SNP as the preferential binding score (PBS) [35]. Spearman’s correlation coefficient was calculated between the ΔMOCCS2score and PBS for each ChIP-seq sample of the same TF.

#### Comparison of the ΔMOCCS2score with the ASB SNPs

SNP lists were obtained from the ADASTRA database [36] (https://adastra.autosome.ru/zanthar, Release Susan v3.5.2), which contains ASB events and their corresponding ASB significance across 674 TFs and 337 cell types. ASB significance indicates changes in the TF-binding specificity induced by ASB SNPs. SNPs overlapping the peak regions from the ChIP-seq samples were selected and *k*-mers overlapping SNPs from the reference genome were obtained (hg38) (ref-*k*-mer). After obtaining the alt*-k-*mer corresponding to the ref*-k-*mer, the AUC score, count, and MOCCS2score were determined from the MOCCS profile and the ΔMOCCS2score, *p*-value, and q-value corresponding to each *k*-mer pair were calculated (ref*-k*-mer and alt*-k*-mer). A large negative ASB significance indicated a strong influence on TF binding caused by a change from the reference allele to the alternative allele.

To compare the ΔMOCCS2score and ASB significance, concordant SNPs between the ΔMOCCS2score and ASB significance were defined as those satisfying the following conditions: (1) ΔMOCCS2score was significant (q < 0.05); (2) |ASB significance| was significant (FDR < 0.05); and (3) the direction of change induced by the SNP was the same between the ΔMOCCS2score and ASB significance. Based on this definition, the ratio of concordant SNPs and discordant SNPs was calculated for each TF. In addition, a permutation test was performed on the percentage of concordant SNPs for each TF by shuffling ΔMOCCS2score profiles 100 times, obtaining the empirical null distribution of the ratio of concordant SNPs and calculating the *p*-value of the observed ratio. Furthermore, the fold change of PWM was obtained from the ADASTRA database and Spearman's correlation between the PWM motif fold-change and the ΔMOCCS2score was calculated.

#### Evaluation of the ΔMOCCS2scores of GWAS-SNPs

GWAS-SNP data was obtained from the GWAS catalog (https://www.ebi.ac.uk/gwas/) [60] for IBD (EFO_0003767), CD (EFO_0000384), MS (EFO_0003885), and SLE (EFO_0002690).

After selecting the SNPs that overlapped with the peaks of the ChIP-seq samples, *k*-mers that overlapped with the SNPs from the reference genome were obtained (hg38) (ref-*k*-mer*)*. After obtaining the alt-*k*-mer by substituting one nucleotide in the ref-*k*-mer, the ΔMOCCS2score was calculated with a *p*-value and q-value for each ref*-k*-mer and alt-*k*-mer pair. SNPs whose ΔMOCCS2scores were not calculated were excluded because repeat and low-confidence regions in the reference genome had been removed from the analyses.

The number of peak-overlapping or out-of-peak SNPs was counted and the ratio of SNPs with significant ΔMOCCS2scores was calculated (q < 0.05) for each phenotype.

The association between allele frequency and absolute values of the ΔMOCCS2score or the ratio of SNPs with a significant ΔMOCCS2score was tested using linear regression. The (1) allele frequency or (2) rank of allele frequency after categorization was set into five bins as an explanatory variable and (1) the absolute values of the ΔMOCCS2score or (2) the ratio of SNPs with a significant ΔMOCCS2score was set as a response. The *p*-values of the regression coefficients were calculated using F-tests.

#### Statistical tests

The statistical tests used for each respective purpose are as follows:

- Wilcoxon signed-rank test (as a non-parametric test for paired two-group comparison of non-Gaussian data): Fig. 2D, E
- Mann–Whitney U test (as a non-parametric test for unpaired two-group comparison): Figs. 2G, 3B, D
- One sample t-test using the asymptotic t approximation (as a test of whether an observed Spearman correlation coefficient is significantly different from zero; Implemented in the ‘cor.test()’ function in the R ‘stats’ package): Figures S4C, S5C, 5C
- Permutation test (as a test of whether an observed value was significantly high in the given complex data structures): Figs. 2I, 3C, 5E
- Two-sided Chi-squared test for difference of two proportions: Tables S3 and S4

### Supplementary Information

**Additional file 1: Figure S1** Filtering of ChIP-seq samples. A: Schematic overview of ChIP-seq sample filtering. B: Violin plot showing the AUROC of the prediction of the top 10% PWM-supported *k*-mers based on the MOCCS2score. The red violin plot represents all CTCF ChIP-seq samples, the green plot represents soft-filtered CTCF ChIP-seq samples, and the blue plot represents hard-filtered CTCF ChIP-seq samples. High-quality ChIP-seq samples with high AUROC scores were retained after hard filtering. C: Distribution of each quality control metric of ChIP-seq sample filtering for samples that passed the hard filter (pink) and others (blue). D: Bar plots display the number of ChIP-seq samples that passed through the soft and hard filters. Bars are colored according to cell type classes or TFs. **Figure S2** Simulation of significant *k*-mer detection. A: The procedure for generating simulated datasets. Simulated data generated by embedding a “true significant *k*-mer” within random sequences was applied to MOCCS2 and the q-values of the MOCCS2score were calculated for each *k*-mer. B: Parameters for each simulation condition from #1 to #5. α is the percentage of input sequences containing embedded “true significant *k*-mers” , N is the number of peaks in a ChIP-seq sample, and σ is the standard deviation of the embedded “true significant *k*-mers” from the center of the peak. C: Simulation results for significant *k*-mer detection. The sensitivity, specificity, and FDR for detecting “true significant *k*-mers” are shown for different parameter settings. **Figure S3** Number of peaks and significant *k*-mers in MOCCS profiles. A: Number of peaks in MOCCS profiles. The x-axis represents the log-transformed number of peaks with a base of 10 and the y-axis represents the number of ChIP-seq samples. B: Relationship between the number of peaks and significant *k*-mers in MOCCS profiles (left, q < 0.05; right, q < 0.01). **Figure S4** Similarities in MOCCS profiles and peak locations for sample pairs of same or different TFs. A: Comparison of *k*-sim Jaccard, Pearson and peak overlap indices (a-c: groups of the same cell types). B: Two-dimensional density plot of *k*-sim Jaccard or Pearson with the peak overlap index (a-c: groups of the same cell types). C: Correlation coefficient of *k*-sim Jaccard or Pearson with the peak overlap index in each group. The y-axis indicates Spearman’ s correlation coefficient. Red and blue indicate *k*-sim Pearson and Jaccard values, respectively (a-c: groups of the same cell types) **Figure S5** Similarities in MOCCS profiles and peak locations for sample pairs of same/different cell types. A: Comparison of the *k*-sim Jaccard, Pearson, and peak overlap indices (a, d, and e: groups of the same TFs). B: Two-dimensional density plot of *k*-sim Jaccard or Pearson with the peak overlap index (a, d, and e: groups of the same TFs). C: Correlation coefficient of *k*-sim Jaccard or Pearson with the peak overlap index in each group. The y-axis indicates Spearman’ s correlation coefficient. Red and blue indicate *k*-sim Pearson and Jaccard values, respectively (a, d, and e: groups of the same TFs). **Figure S6** Heat maps of cell type-dependent TFs. The heat map color indicates the *k*-sim Jaccard value for the 33 cell type-dependent TFs. The color labels of the heat maps indicate the cell type classes. Cell type classes with only a single ChIP-seq sample were excluded from the visualization. Asterisks indicate the statistical significance of ChIP-seq samples with the same and different cell type classes (Mann–Whitney U test, p < 0.05). **Figure S7** Violin plots of all cell type-dependent TFs. The y-axis indicates the *k*-sim Jaccard value. The same and different groups were arranged along the x-axis. Asterisks indicate the statistical significance of ChIP-seq samples with the same and different cell type classes (Mann–Whitney U test, p < 0.05). **Figure S8** Simulation of differential *k*-mer detection. A: Simulated data processing. Simulated data with an embedded “true differential *k*-mer” and “true significant *k*-mer” was prepared by embedding a “true” *k*-mer within *α*% of a randomly generated sample of 2*W *+ 1 bp (*W *= 350) DNA sequences and applied to MOCCS2. “True significant *k*-mers” were embedded following a normal distribution whose mean was *W *+ 1 and whose standard deviation was *σ*. “True differential *k*-mers” were embedded in S1 (or S2), similar to “true significant *k*-mers,” and were embedded in S2 (or S1) following a uniform distribution whose mean was 1 and whose standard deviation was (2 × *W *+ 1) − (*k *− 1). It should be noted that we set *k *as *k*=6. B: Parameters for each simulation condition from #1 to #5. *L *is the number of differential *k*-mers and m is the number of significant *k*-mers. **Figure S9** ΔMOCCS2score profiles were consistent with the in vitro SNP-SELEX and PWM motif fold change. A: Spearman’ s correlation coefficient between PBS (SNP-SELEX) and ΔMOCCS2score in each TF for the original and permuted data. Red points indicate the original Spearman’ s correlation coefficient, and blue points indicate the permutated data. B: Difference in ΔMOCCS2score profile consistency among the positions of SNPs in *k*-mers. The *k*th SNP position indicates the *k*th allele on the left side of the *k*-mer. C: The ΔMOCCS2score is consistent with the PWM motif fold change. **Figure S10** Number of peak-overlapping GWAS-SNPs with significant ΔMOCCS2scores. Number of peak-overlapping GWAS-SNPs in each ChIP-seq sample. Each bar represents a ChIP-seq sample, and the y-axis represents the number of peak-overlapping GWAS-SNPs. The red fraction represents the number of peak-overlapping GWAS-SNPs with significant ΔMOCCS2scores (q < 0.05), and the gray fraction represents the number of GWAS SNPs with non-significant ΔMOCCS2scores. **Figure S11** Prediction of SNP-affected TFs and cell type classes using ΔMOCCS2score profiles. Top ChIP-seq samples with high ΔMOCCS2scores in each phenotype (IBD, inflammatory bowel disease; CD, Crohn’ s disease; MS, multiple sclerosis; SLE, systemic lupus erythematosus). The ΔMOCCS2score was calculated for each SNP and ChIP-seq sample. Bar graph colors represent TFs or cell type classes. **Figure S12** Association between the allele frequency and ΔMOCCS2score. Association between the allele frequency and (A) the absolute values of the ΔMOCCS2score or (B) the ratio of SNPs with significant ΔMOCCS2scores in each phenotype (IBD, inflammatory bowel disease; CD, Crohn’ s disease; MS, multiple sclerosis; SLE, systemic lupus erythematosus). **Figure S13** Accuracy of detecting canonical motifs using MOCCS2score for different *k*. AUROC for detecting canonical PWM motifs using the MOCCS2score in the difference of value *k*. The x-axis represents the ratio of PWM-supported *k*-mers in all *k*-mers and the y-axis represents the AUROC. The colors of the violin plots represent the different *k *values.

**Additional file 2: Supplementary Table 1.** List of cell type-dependent TFs.

**Additional file 3: Supplementary Table 2.** List of cell type-dependent TFs and cell types.

**Additional file 4: Supplementary Table 3.** Number of cell type-dependent TFs in each TF family.

**Additional file 5: Supplementary Table 4.** Number of cell type-dependent TFs in each Cell-type-class pair.

## Data Availability

All human datasets used in the study are publicly available. The human TF ChIP-seq data were obtained from the ChIP-Atlas database (https://chip-atlas.org/) [22]. The SNP-SELEX results were obtained from the Gene Expression Omnibus under the accession number GSE118725 [35]. The allele-specific binding SNP lists were obtained from the ADASTRA database (https://adastra.autosome.ru/zanthar, Release Susan v3.5.2) [36]. The GWAS-SNP lists was obtained from the GWAS catalog (https://www.ebi.ac.uk/gwas/) [60] for IBD (EFO_0003767), CD (EFO_0000384), MS (EFO_0003885), and SLE (EFO_0002690).The codes and data are available on the GitHub repository (https://github.com/bioinfo-tsukuba/MOCCS_paper_public). The following files are available on the figshare repository (DOI:10.6084/m9.figshare.19333646): (1) SNPs with a significant PBSscore overlapping the ΔMOCCS2score profile (SELEX_dMOCCS2score.tsv); (2) SNPs with ASBs overlapping the ΔMOCCS2score profile (ASB_dMOCCS2score.tsv); (3) GWAS-SNP overlapping the ΔMOCCS2score profile (GWAS_dMOCCS2score.tsv).

## Abbreviations

ASB: Allele-specific binding
AUROC: Area Under Receiver Operating Characteristic Curve
ChIP-seq: Chromatin immunoprecipitation sequencing
GWAS: Genome-wide association study
PBS: Preferential binding score
PWM: Position weight matrix
SNP: Single-nucleotide polymorphism
TF: Transcription factor
TFBS: Transcription factor binding site
MOCCS: Motif centrality analysis of ChIP-seq
