# Supplementary material for: Transcription factor-binding k-mer analysis clarifies the cell type dependency of binding specificities and cis-regulatory SNPs in humans
Source: BMC Genomics. 2023 Oct 7;24:597. doi: 10.1186/s12864-023-09692-9 (PMC10560430; doi:10.1186/s12864-023-09692-9)
Supplement: Supplementary file 1 — Additional file 1: Figure S1 Filtering of ChIP-seq samples. A: Schematic overview of ChIP-seq sample filtering. B: Violin plot showing the AUROC of the prediction of the top 10% PWM-supported k-mers based on the MOCCS2score. The red violin plot represents all CTCF ChIP-seq samples, the green plot represents soft-filtered CTCF ChIP-seq samples, and the blue plot represents hard-filtered CTCF ChIP-seq samples. High-quality ChIP-seq samples with high AUROC scores were retained after hard filtering. C: Distribution of each quality control metric of ChIP-seq sample filtering for samples that passed the hard filter (pink) and others (blue). D: Bar plots display the number of ChIP-seq samples that passed through the soft and hard filters. Bars are colored according to cell type classes or TFs. Figure S2 Simulation of significant k-mer detection. A: The procedure for generating simulated datasets. Simulated data generated by embedding a “true significant k-mer” within random sequences was applied to MOCCS2 and the q-values of the MOCCS2score were calculated for each k-mer. B: Parameters for each simulation condition from #1 to #5. α is the percentage of input sequences containing embedded “true significant k-mers” , N is the number of peaks in a ChIP-seq sample, and σ is the standard deviation of the embedded “true significant k-mers” from the center of the peak. C: Simulation results for significant k-mer detection. The sensitivity, specificity, and FDR for detecting “true significant k-mers” are shown for different parameter settings. Figure S3 Number of peaks and significant k-mers in MOCCS profiles. A: Number of peaks in MOCCS profiles. The x-axis represents the log-transformed number of peaks with a base of 10 and the y-axis represents the number of ChIP-seq samples. B: Relationship between the number of peaks and significant k-mers in MOCCS profiles (left, q < 0.05; right, q < 0.01). Figure S4 Similarities in MOCCS profiles and peak locations for sample pairs of same [file 12864_2023_9692_MOESM1_ESM.pdf]

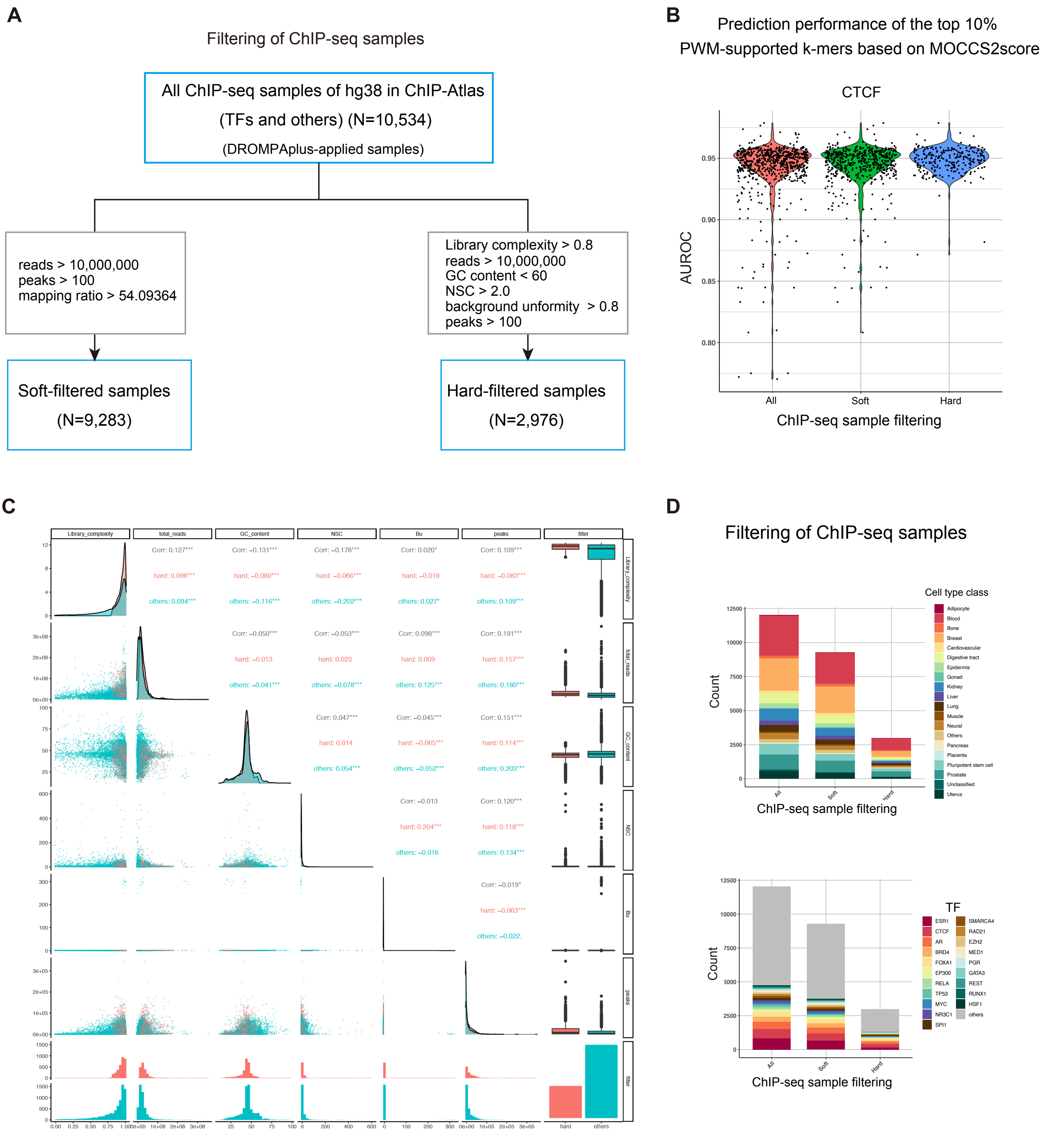

Figure S1 Filtering of ChIP-seq samples. A: Schematic overview of ChIP-seq sample filtering. B. Violin plot showing the AUROC of the prediction of the top 10% PWM-supported *k*-mers based on the MOCCS2score. The red violin plot represents all CTCF ChIP-seq samples, the green plot represents soft-filtered CTCF ChIP-seq samples, and the blue plot represents hard-filtered CTCF ChIP-seq samples. High-quality ChIP-seq samples with high AUROC scores were retained after hard filtering. C: Distribution of each quality control metric of ChIP-seq sample filtering for samples that passed the hard filter (pink) and others (blue). D. Bar plots display the number of ChIP-seq samples that passed through the soft and hard filters. Bars are colored according to cell type classes or TFs.

Tahara *et al.*, Figure S1

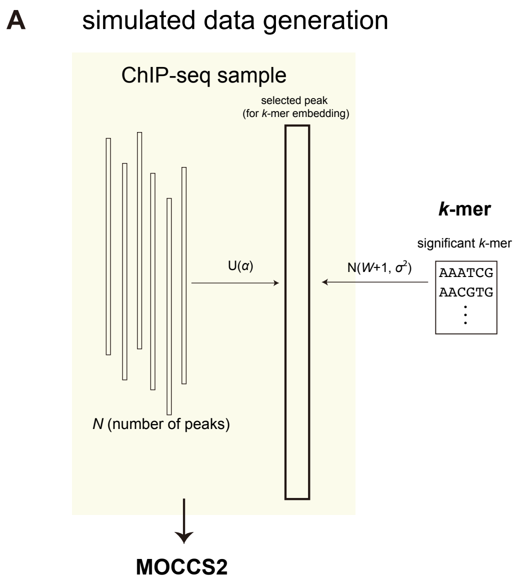

B Parameters for simulation (significant *k*-mer)

simulation	*α*	*N*	*σ*
#1	[0.1, 0.2]	12,000	*W*/5
#2	[0.01, 0.05]	12,000	*W*/5
#3	[0.05, 0.1]	12,000	*W*/5
#4	[0.1, 0.2]	6,000	*W*/5
#5	[0.1, 0.2]	12,000	*W*/2

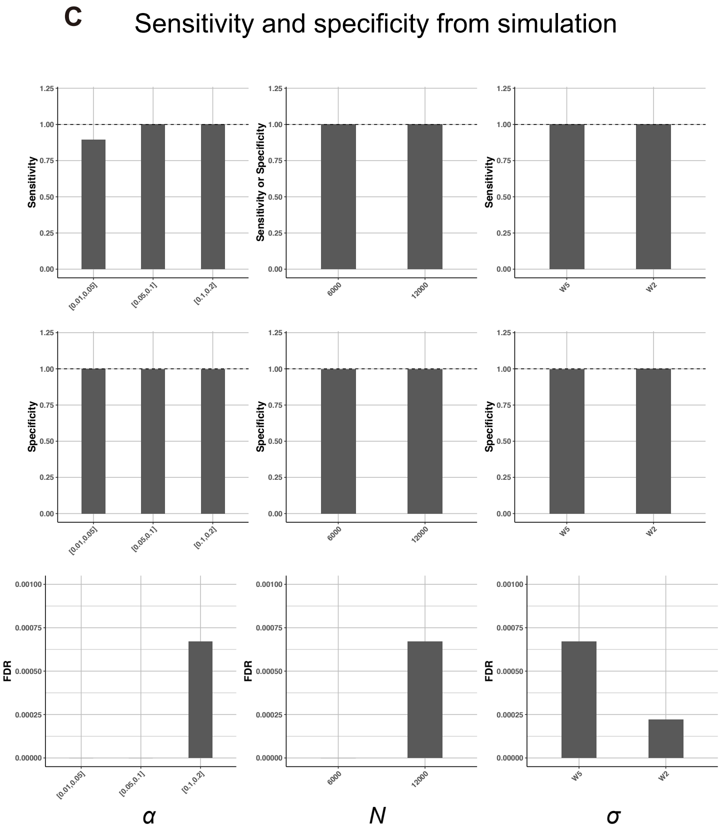

Figure S2 Simulation of significant *k*-mer detection. A: The procedure for generating simulated datasets. Simulated data generated by embedding a "true significant *k*-mer" within random sequences was applied to MOCCS2 and the q-values of the MOCCS2score were calculated for each *k*-mer. B: Parameters for each simulation condition from #1 to #5. α is the percentage of input sequences containing embedded "true significant *k*-mers" , N is the number of peaks in a ChIP-seq sample, and σ is the standard deviation of the embedded "true significant *k*-mers" from the center of the peak. C: Simulation results for significant *k*-mer detection. The sensitivity, specificity, and FDR for detecting "true significant *k*-mers" are shown for different parameter settings.

Tahara *et al*., Figure S2

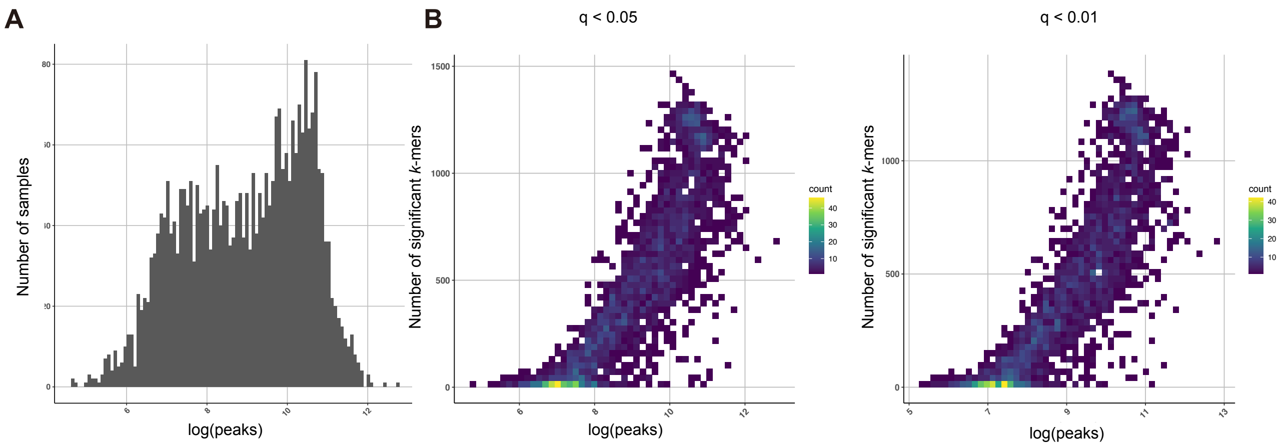

Figure S3 Number of peaks and significant *k*-mers in MOCCS profiles. A: Number of peaks in MOCCS profiles. The x-axis represents the log-transformed number of peaks with a base of 10 and the y-axis represents the number of ChIP-seq samples. B: Relationship between the number of peaks and significant *k*-mers in MOCCS profiles (left, $q < 0.05$; right, $q < 0.01$).

Tahara *et al*., Figure S3

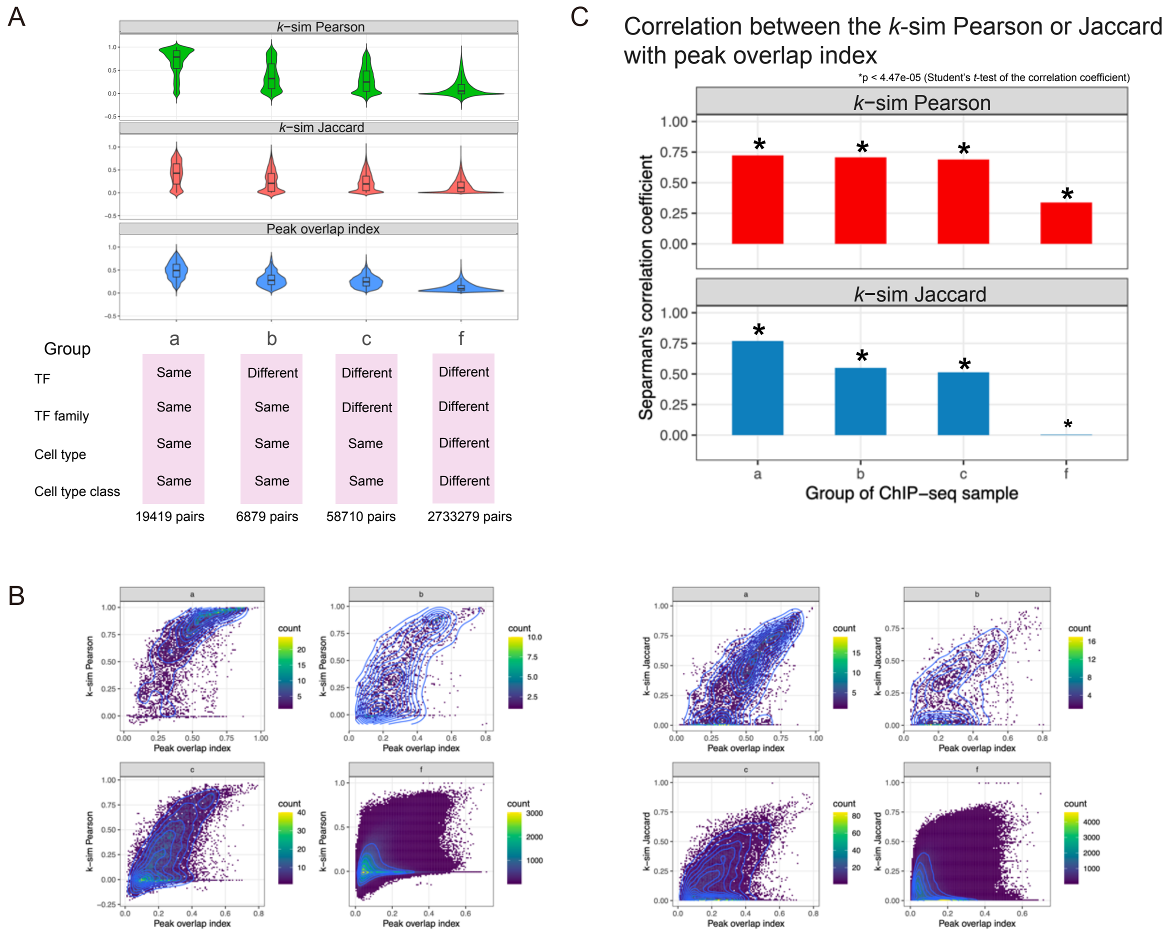

Figure S4 Similarities in MOCCS profiles and peak locations for sample pairs of same or different TFs. A: Comparison of *k*-sim Jaccard, Pearson and peak overlap indices (a-c: groups of the same cell types). B: Two-dimensional density plot of *k*-sim Jaccard or Pearson with the peak overlap index (a-c: groups of the same cell types). C: Correlation coefficient of *k*-sim Jaccard or Pearson with the peak overlap index in each group. The y-axis indicates Spearman’ s correlation coefficient. Red and blue indicate *k*-sim Pearson and Jaccard values, respectively (a-c: groups of the same cell types)

.

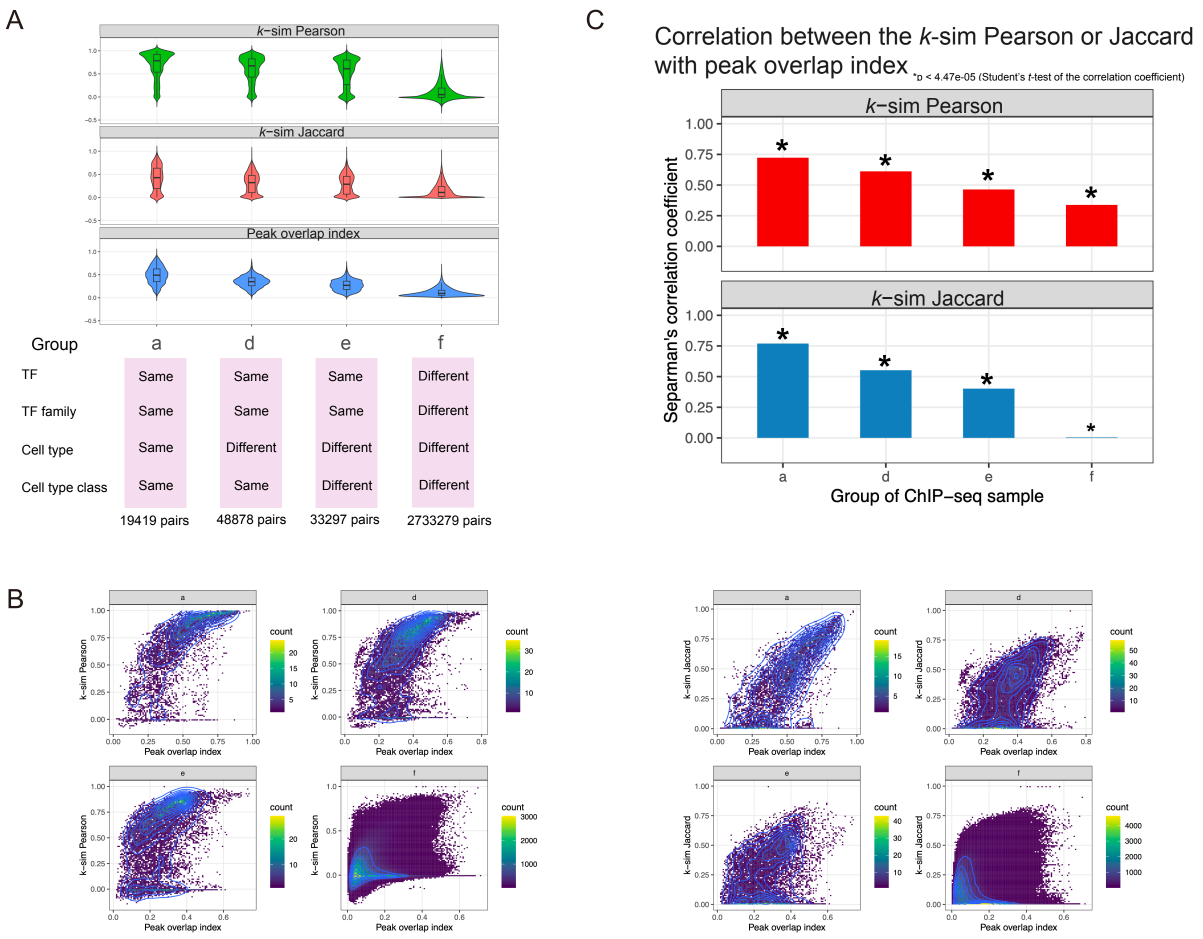

Figure S5 Similarities in MOCCS profiles and peak locations for sample pairs of same/different cell types. A: Comparison of the *k*-sim Jaccard, Pearson, and peak overlap indices (a, d, and e: groups of the same TFs). B: Two-dimensional density plot of *k*-sim Jaccard or Pearson with the peak overlap index (a, d, and e: groups of the same TFs). C: Correlation coefficient of *k*-sim Jaccard or Pearson with the peak overlap index in each group. The y-axis indicates Spearman’ s correlation coefficient. Red and blue indicate *k*-sim Pearson and Jaccard values, respectively (a, d, and e: groups of the same TFs).

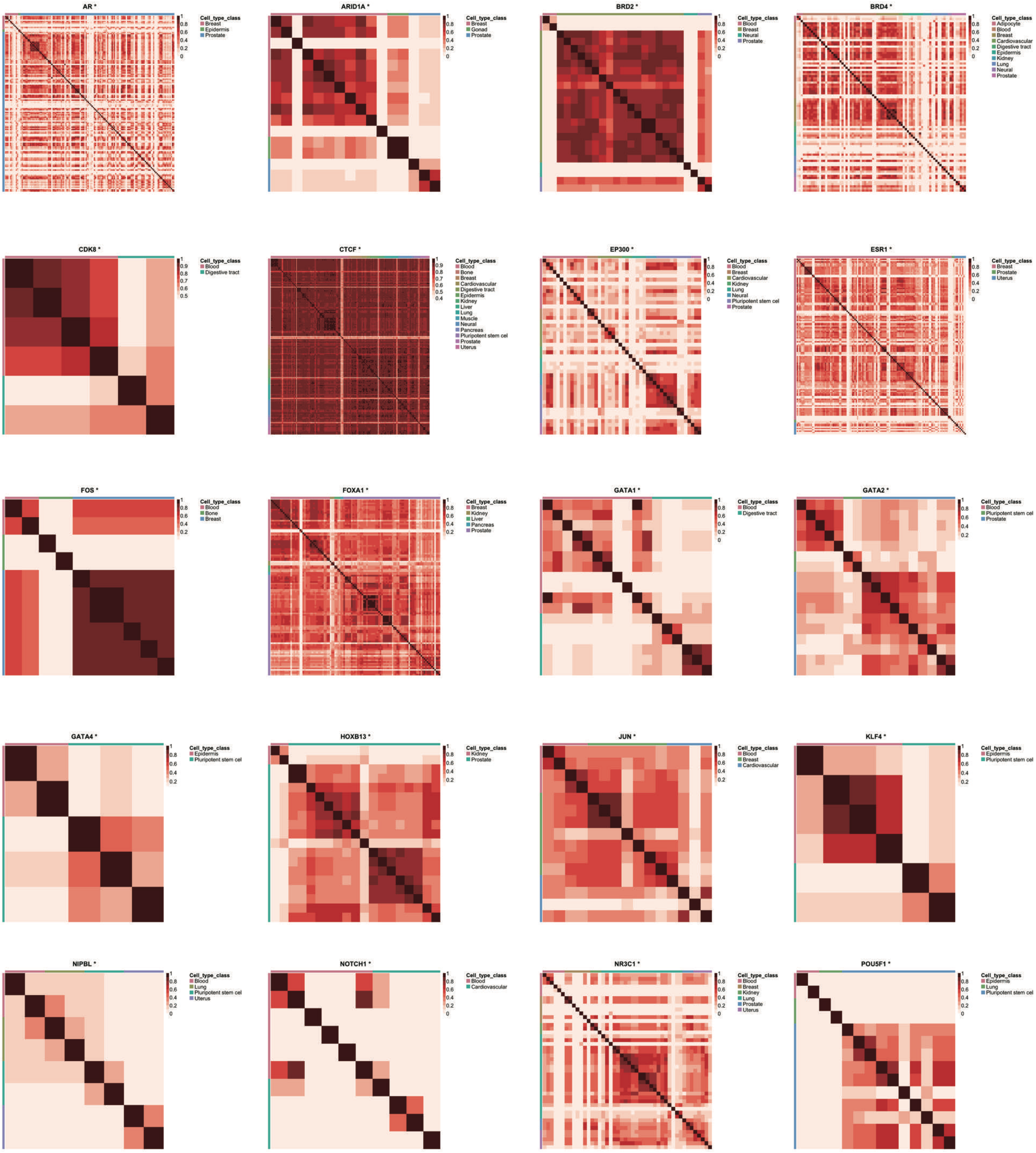
AR *
Cell_type_class
Breast
Epidermis
Prostate
ARID1A *
Cell_type_class
Breast
Gonad
Prostate
BRD2 *
Cell_type_class
Blood
Breast
Neural
Prostate
BRD4 *
Cell_type_class
Adipocyte
Blood
Breast
Cardiovascular
Digestive tract
Epidermis
Kidney
Lung
Neural
Prostate
CDK8 *
Cell_type_class
Blood
Digestive tract
CTCF *
Cell_type_class
Blood
Bone
Breast
Cardiovascular
Digestive tract
Epidermis
Kidney
Liver
Lung
Muscle
Neural
Pancreas
Pluripotent stem cel
Prostate
Uterus
EP300 *
Cell_type_class
Blood
Breast
Cardiovascular
Kidney
Lung
Neural
Pluripotent stem cel
Prostate
ESR1 *
Cell_type_class
Breast
Prostate
Uterus
FOS *
Cell_type_class
Blood
Bone
Breast
FOXA1 *
Cell_type_class
Breast
Kidney
Liver
Pancreas
Prostate
GATA1 *
Cell_type_class
Blood
Digestive tract
GATA2 *
Cell_type_class
Blood
Pluripotent stem cel
Prostate
GATA4 *
Cell_type_class
Epidermis
Pluripotent stem cel
HOXB13 *
Cell_type_class
Kidney
Prostate
JUN *
Cell_type_class
Blood
Breast
Cardiovascular
KLF4 *
Cell_type_class
Epidermis
Pluripotent stem cel
NIPBL *
Cell_type_class
Blood
Lung
Pluripotent stem cel
Uterus
NOTCH1 *
Cell_type_class
Blood
Cardiovascular
NR3C1 *
Cell_type_class
Blood
Breast
Kidney
Lung
Prostate
Uterus
POU5F1 *
Cell_type_class
Epidermis
Lung
Pluripotent stem cel

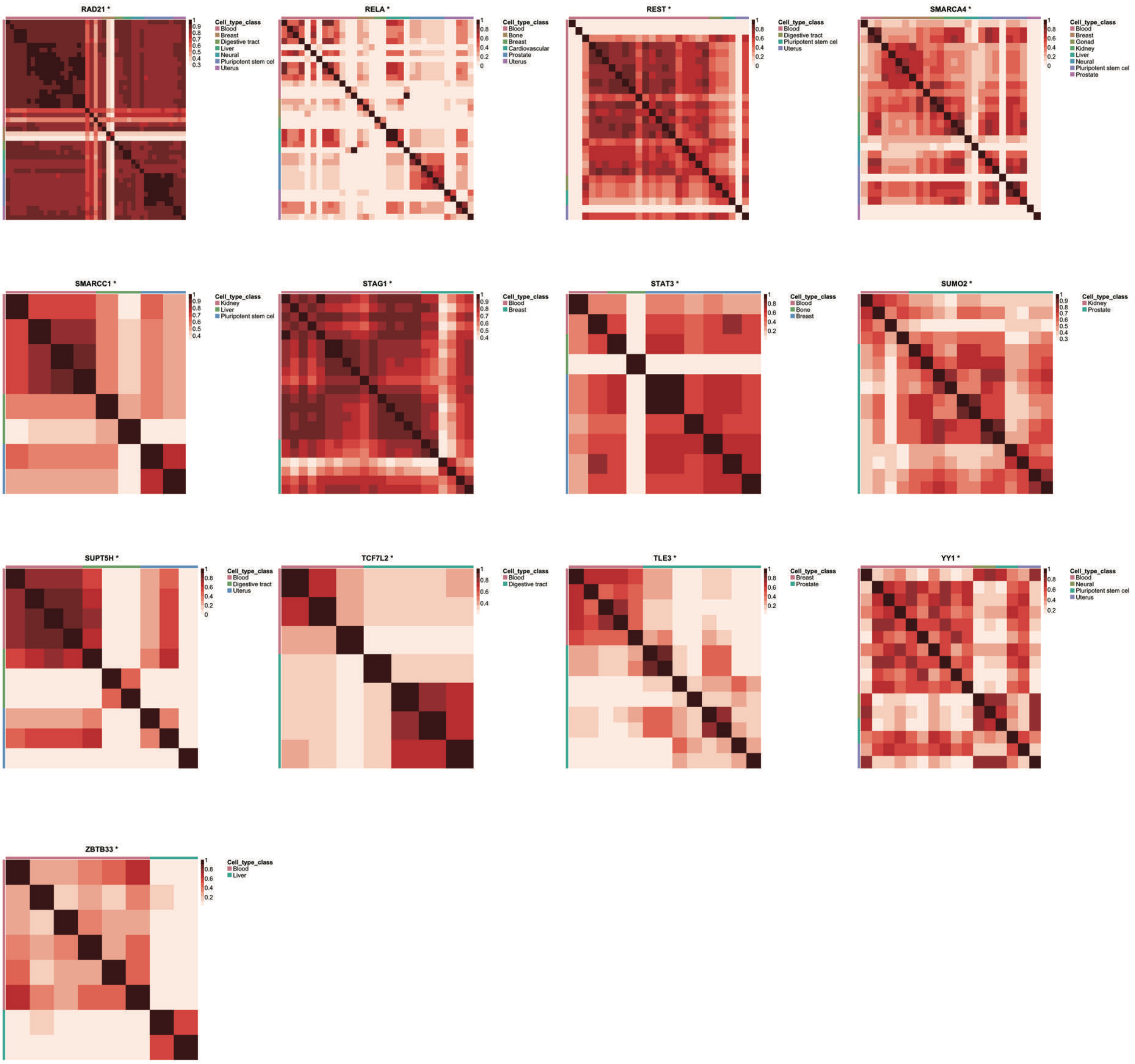

Figure S6 Heat maps of cell type-dependent TFs. The heat map color indicates the *k*-sim Jaccard value for the 33 cell type-dependent TFs. The color labels of the heat maps indicate the cell type classes. Cell type classes with only a single ChIP-seq sample were excluded from the visualization. Asterisks indicate the statistical significance of ChIP-seq samples with the same and different cell type classes (Mann–Whitney U test, $p < 0.05$).

Tahara et al., Figure S6

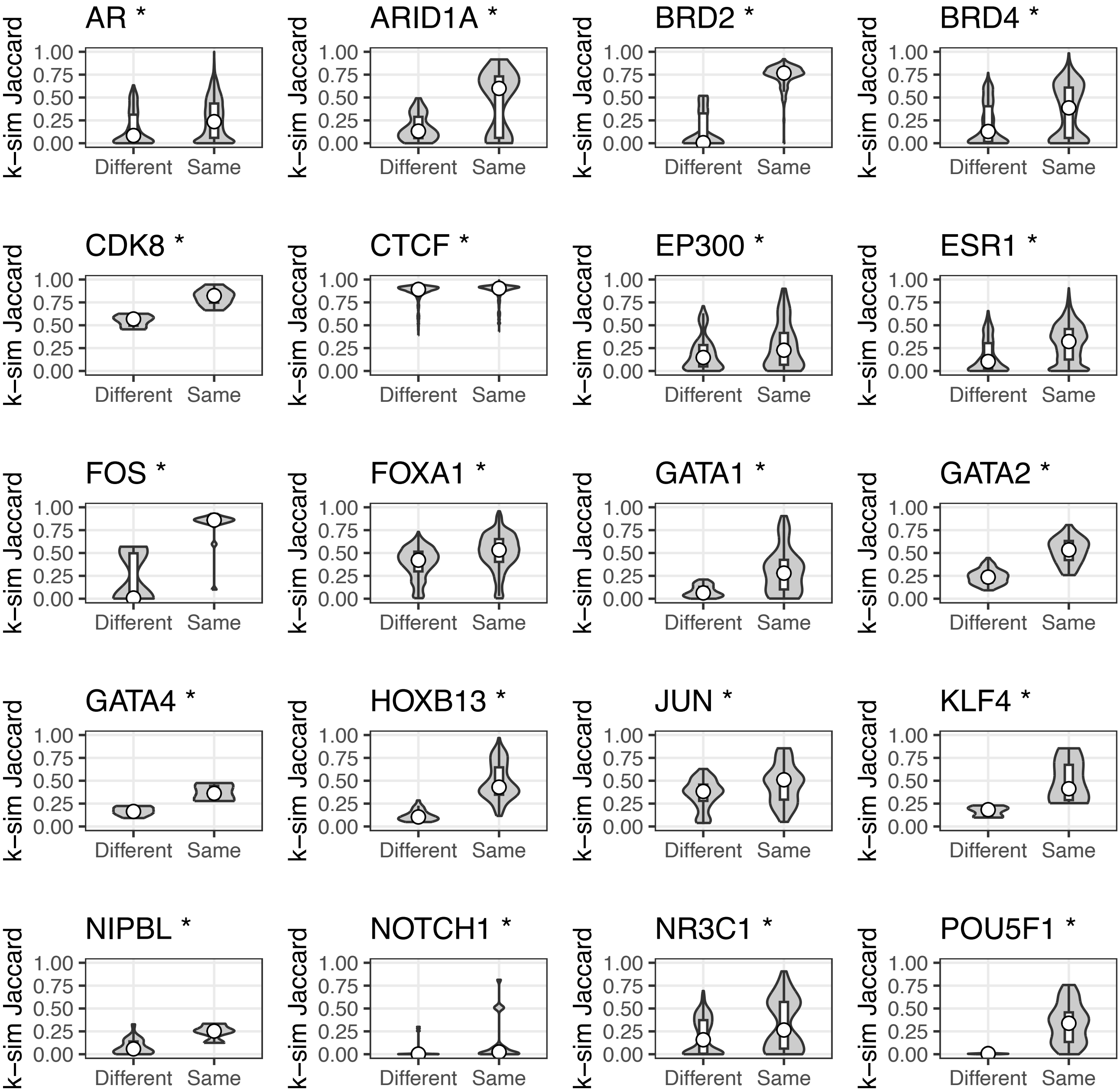

AR *
ARID1A *
BRD2 *
BRD4 *
CDK8 *
CTCF *
EP300 *
ESR1 *
FOS *
FOXA1 *
GATA1 *
GATA2 *
GATA4 *
HOXB13 *
JUN *
KLF4 *
NIPBL *
NOTCH1 *
NR3C1 *
POU5F1 *
k−sim Jaccard
1.00
0.75
0.50
0.25
0.00
Different
Same

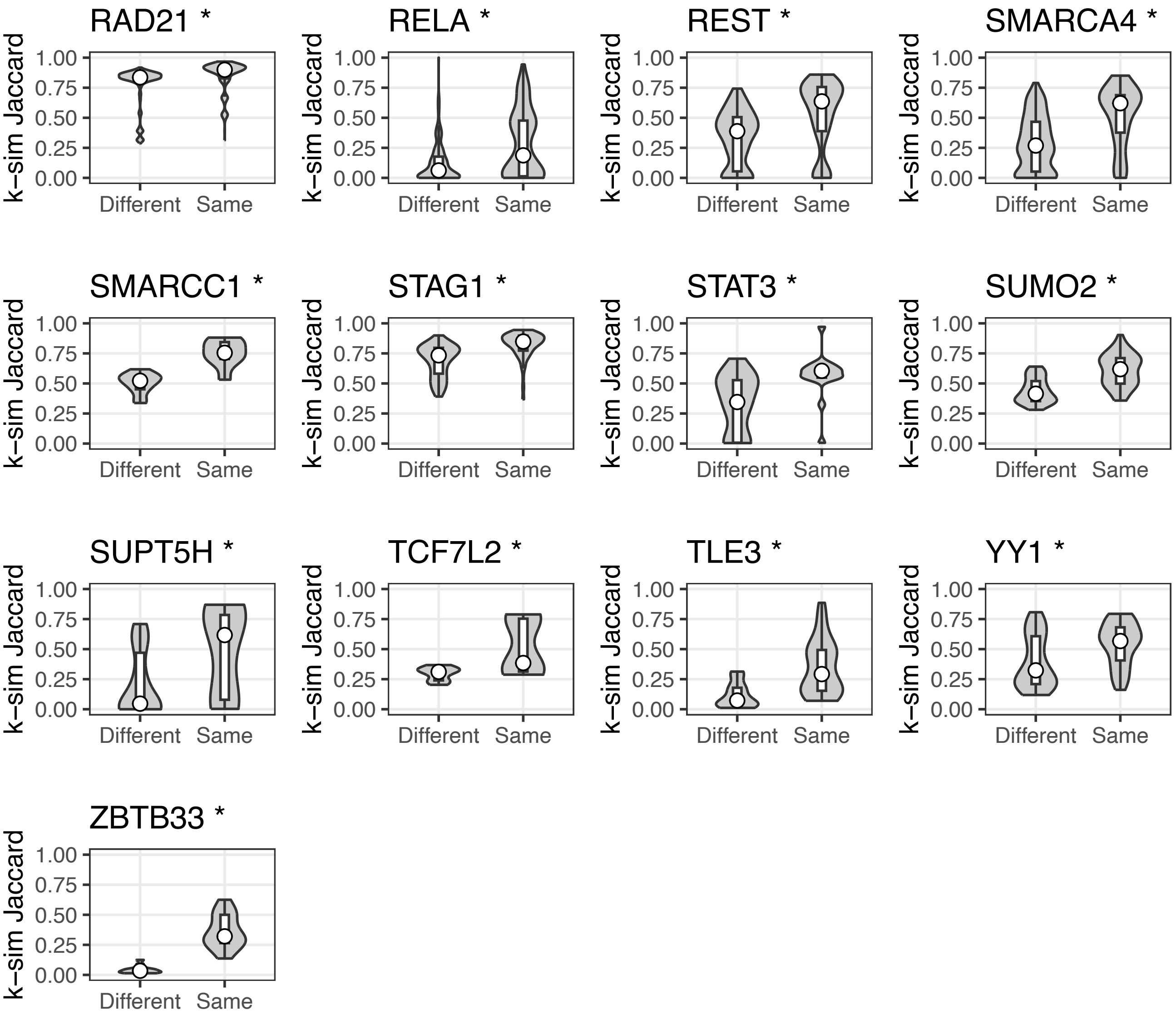

Figure S7 Violin plots of all cell type-dependent TFs. The y-axis indicates the *k*-sim Jaccard value. The same and different groups were arranged along the x-axis. Asterisks indicate the statistical significance of ChIP-seq samples with the same and different cell type classes (Mann–Whitney U test, $p < 0.05$).

A

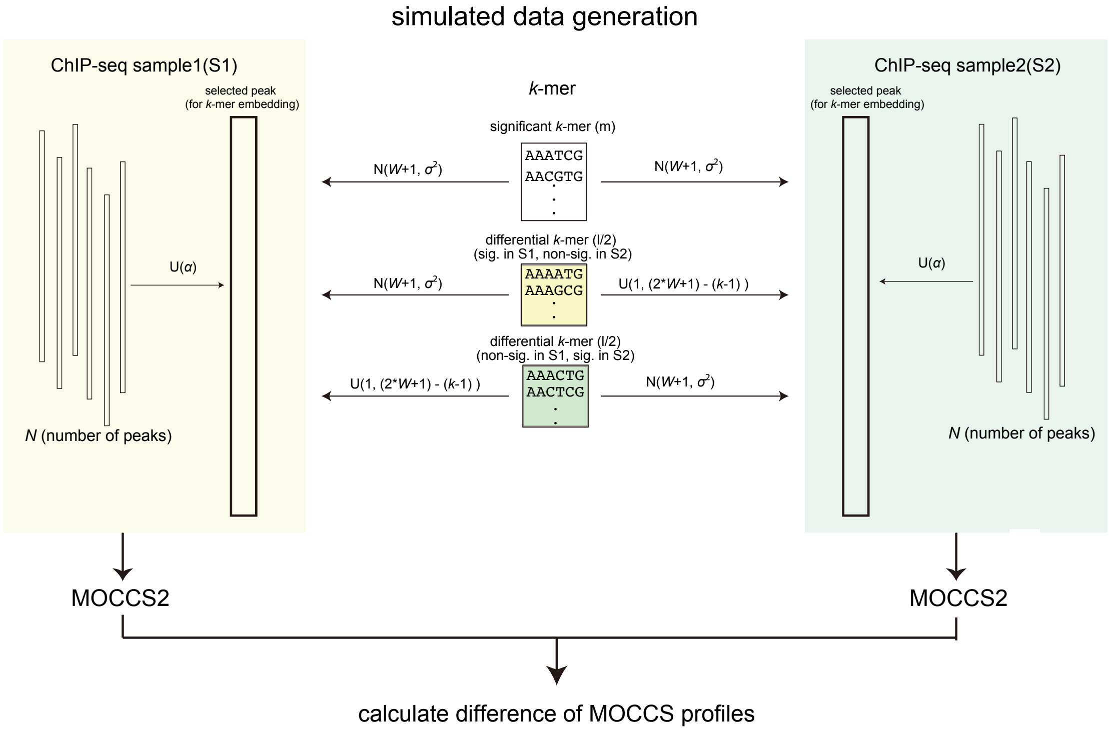

B

simulation	α	N	σ
#1	[0.10, 0.20]	12000	W/5
#2	[0.01, 0.05]	12000	W/5
#3	[0.05, 0.10]	12000	W/5
#4	[0.10, 0.20]	6000	W/5
#5	[0.10, 0.20]	12000	W/2

α : The percentage of the input sequences containing embedded "true significant *k*-mers"

N : The number of peaks in a ChIP-seq sample

σ : The standard deviation of the embedded "true significant *k*-mers" from the center of the peak

W: 2*W*+1bp means a length of a peak in a ChIP-seq

l : Number of embedded differential *k*-mers

m : Number of embedded significant *k*-mers

Figure S8 Simulation of differential *k*-mer detection. A: Simulated data processing. Simulated data with an embedded "true differential *k*-mer" and "true significant *k*-mer" was prepared by embedding a "true" *k*-mer within α% of a randomly generated sample of $2W + 1$ bp ($W = 350$) DNA sequences and applied to MOCCS2. "True significant *k*-mers" were embedded following a normal distribution whose mean was $W + 1$ and whose standard deviation was σ. "True differential *k*-mers" were embedded in S1 (or S2), similar to "true significant *k*-mers," and were embedded in S2 (or S1) following a uniform distribution whose mean was 1 and whose standard deviation was $(2 \times W + 1) - (k - 1)$. It should be noted that we set k as k=6. B: Parameters for each simulation condition from #1 to #5. L is the number of differential *k*-mers and m is the number of significant *k*-mers.

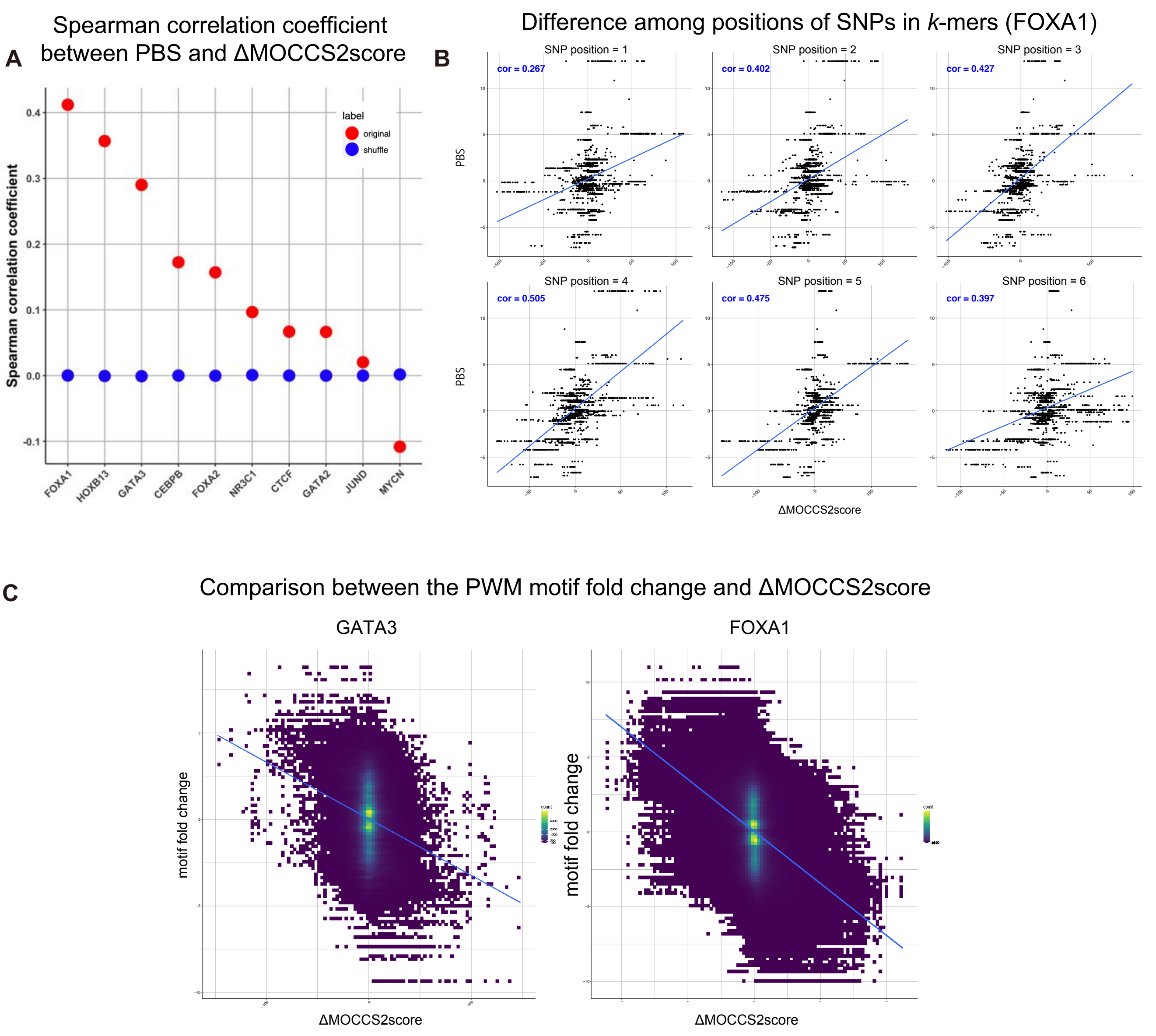

Figure S9 ΔMOCCS2score profiles were consistent with the in vitro SNP-SELEX and PWM motif fold change. A: Spearman’ s correlation coefficient between PBS (SNP-SELEX) and ΔMOCCS2score in each TF for the original and permuted data. Red points indicate the original Spearman’ s correlation coefficient, and blue points indicate the permutated data. B: Difference in ΔMOCCS2score profile consistency among the positions of SNPs in *k*-mers. The *k*th SNP position indicates the *k*th allele on the left side of the *k*-mer. C: The ΔMOCCS2score is consistent with the PWM motif fold change.

Number of peak-overlapping GWAS-SNP and the significance of ΔMOCCS2score

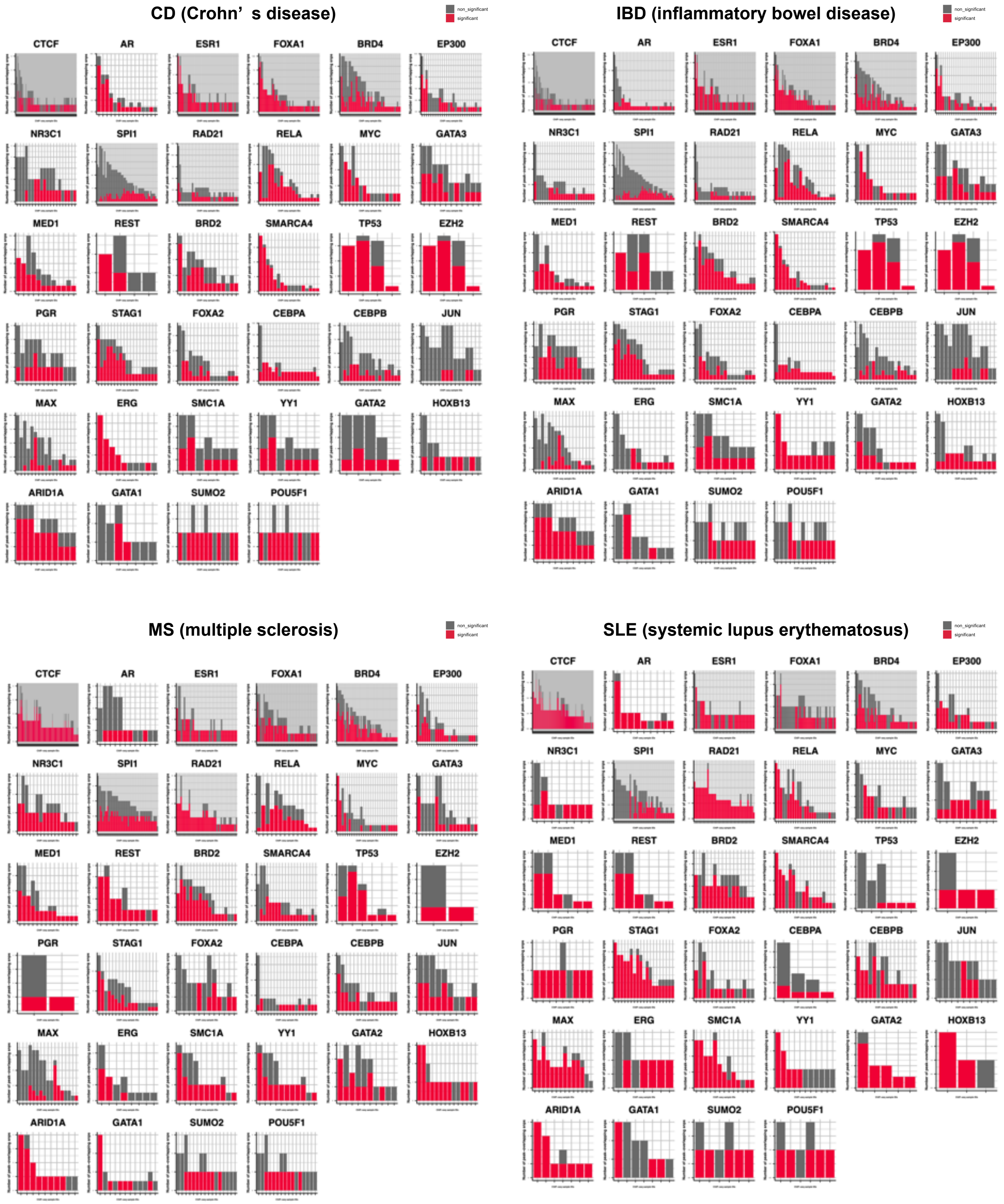

Figure S10 Number of peak-overlapping GWAS-SNPs with significant ΔMOCCS2scores. Number of peak-overlapping GWAS-SNPs in each ChIP-seq sample. Each bar represents a ChIP-seq sample, and the y-axis represents the number of peak-overlapping GWAS-SNPs. The red fraction represents the number of peak-overlapping GWAS-SNPs with significant ΔMOCCS2scores ($q < 0.05$), and the gray fraction represents the number of GWAS SNPs with non-significant ΔMOCCS2scores.

Tahara *et al.*, Figure S10

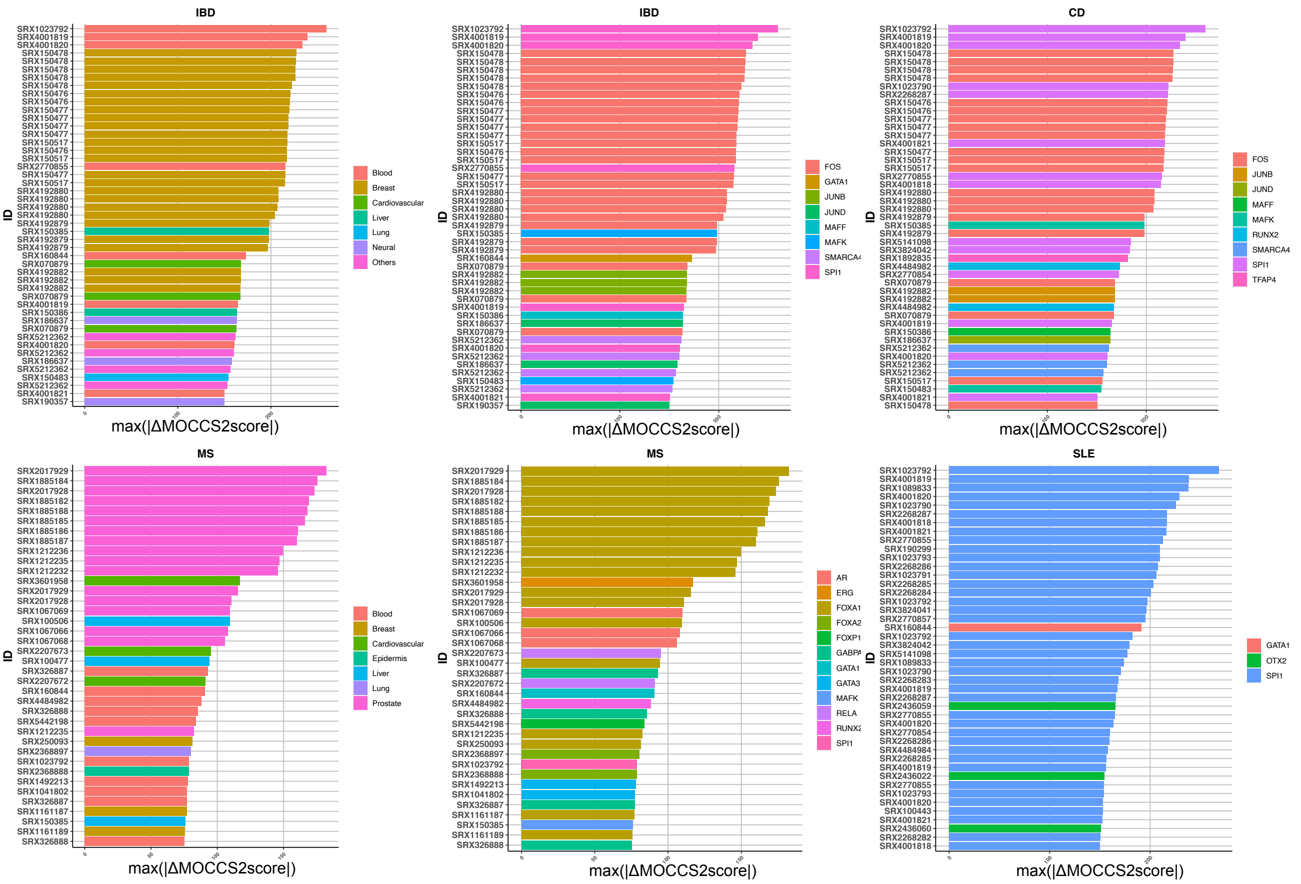

Figure S11 Prediction of SNP-affected TFs and cell type classes using ΔMOCCS2score profiles. Top ChIP-seq samples with high ΔMOCCS2scores in each phenotype (IBD, inflammatory bowel disease; CD, Crohn' s disease; MS, multiple sclerosis; SLE, systemic lupus erythematosus). The ΔMOCCS2score was calculated for each SNP and ChIP-seq sample. Bar graph colors represent TFs or cell type classes.

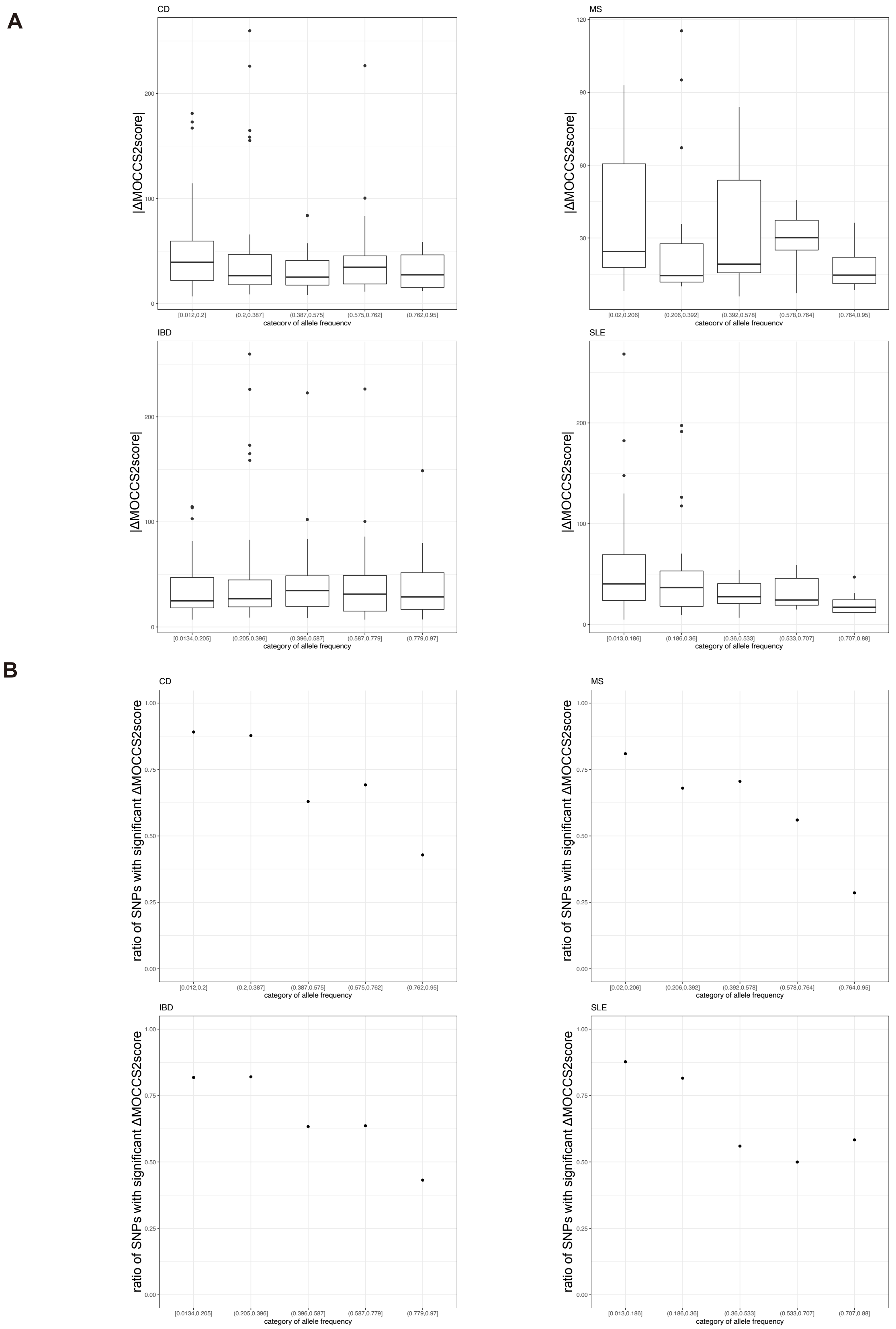

Figure S12 Association between the allele frequency and ΔMOCCS2score. Association between the allele frequency and (A) the absolute values of the ΔMOCCS2score or (B) the ratio of SNPs with significant ΔMOCCS2scores in each phenotype (IBD, inflammatory bowel disease; CD, Crohn’ s disease; MS, multiple sclerosis; SLE, systemic lupus erythematosus).

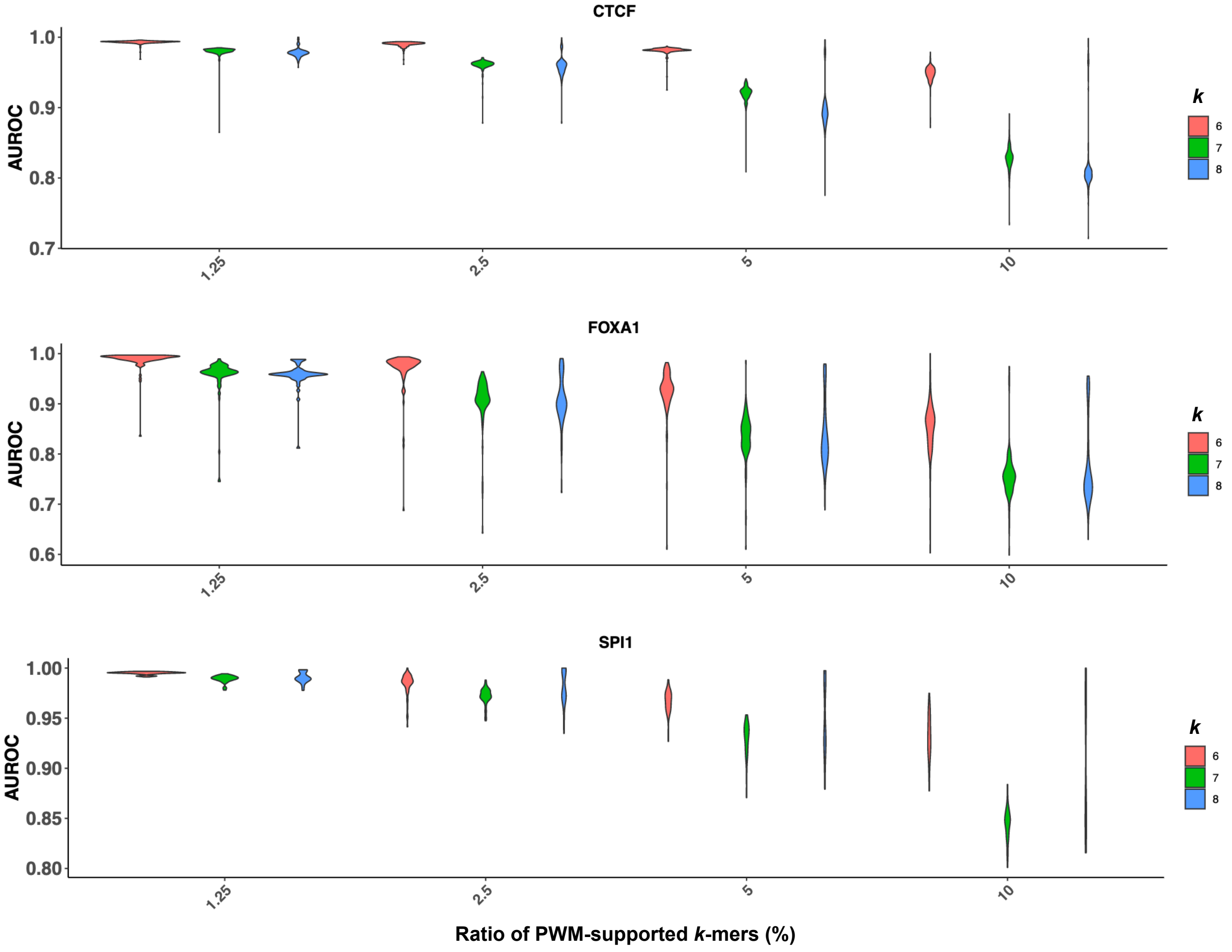

Figure S13 Accuracy of detecting canonical motifs using MOCCS2score for different *k*. AUROC for detecting canonical PWM motifs using the MOCCS2score in the difference of value *k*. The x-axis represents the ratio of PWM-supported *k*-mers in all *k*-mers and the y-axis represents the AUROC. The colors of the violin plots represent the different *k* values.
